# Overexpression of A Biotic Stress-Inducible *Pvgstu* Gene Activates Early Protective Responses in Tobacco under Combined Heat and Drought

**DOI:** 10.3390/ijms22052352

**Published:** 2021-02-26

**Authors:** Evangelia Stavridou, Georgia Voulgari, Michail Michailidis, Stefanos Kostas, Evangelia G. Chronopoulou, Nikolaos E. Labrou, Panagiotis Madesis, Irini Nianiou-Obeidat

**Affiliations:** 1 Laboratory of Genetics and Plant Breeding, School of Agriculture, Forestry and Natural Environment, Aristotle University of Thessaloniki, P.O. Box 261, GR-54124 Thessaloniki, Greece; estavrid@certh.gr (E.S.); georgia.voulgari195@gmail.com (G.V.); 2 Institute of Applied Biosciences, CERTH, 6th km Charilaou-Thermis Road, Thermi, P.O. Box 361, GR-57001 Thessaloniki, Greece; pmadesis@uth.gr; 3 Laboratory of Pomology, Department of Horticulture, School of Agriculture, Aristotle University of Thessaloniki, GR-54124 Thessaloniki, Greece; msmichai@agro.auth.gr; 4 Laboratory of Floriculture, School of Agriculture, Forestry and Natural Environment, Aristotle University of Thessaloniki, GR-54124 Thessaloniki, Greece; skostas@agro.auth.gr; 5 Laboratory of Enzyme Technology, Department of Biotechnology, School of Food, Biotechnology and Development, Agricultural University of Athens, 75 Iera Odos Street, 11855 Athens, Greece; exronop@aua.gr (E.G.C.); lambrou@aua.gr (N.E.L.); 6 Laboratory of Molecular Biology of Plants, School of Agricultural Sciences, University of Thessaly, 38446 Thessaly, Greece

**Keywords:** GSTs, abiotic stress, *P. vulgaris*, *N. tabacum*, morphophysiology, transcriptomics, metabolomics, thermotolerance, primed state

## Abstract

Drought and heat stresses are major factors limiting crop growth and productivity, and their effect is more devastating when occurring concurrently. Plant glutathione transferases (GSTs) are differentially expressed in response to different stimuli, conferring tolerance to a wide range of abiotic stresses. GSTs from drought-tolerant *Phaseolus vulgaris* var. “Plake Megalosperma Prespon” is expected to play an important role in the response mechanisms to combined and single heat and drought stresses. Herein, we examined wild-type *N. tabacum* plants (cv. Basmas Xanthi) and T1 transgenic lines overexpressing the stress-induced *Pvgstu3–3* and *Pvgstu2–2* genes. The overexpression of *Pvgstu3–3* contributed to potential thermotolerance and greater plant performance under combined stress. Significant alterations in the primary metabolism were observed in the transgenic plants between combined stress and stress-free conditions. Stress-responsive differentially expressed genes (DEGs) and transcription factors (TFs) related to photosynthesis, signal transduction, starch and sucrose metabolism, osmotic adjustment and thermotolerance, were identified under combined stress. In contrast, induction of certain DEGs and TF families under stress-free conditions indicated that transgenic plants were in a primed state. The overexpression of the *Pvgstu3–3* is playing a leading role in the production of signaling molecules, induction of specific metabolites and activation of the protective mechanisms for enhanced protection against combined abiotic stresses in tobacco.

## 1. Introduction

Drought and high temperature are two of the most severe environmental factors as they usually occur concurrently, causing a reduction in crop growth and productivity worldwide [1,2], and their combined effect is greater than when occurring individually [3]. Identifying distinctive or common mechanisms necessary for plant acclimatization and tolerance under combined abiotic stresses is a prerequisite for mitigating the negative effects of climate change.

The molecular and morphophysiological responses of plants to drought and heat stress combination are considered unique and cannot be directly extrapolated from the response of plants exposed to either a single drought or heat stress [4,5,6]. Various sensors involved in systemic responses to stress are transduced by metabolic and other signaling pathways, including transcription factors, photosynthetic mechanisms, antioxidant mechanisms, pathogen responses, hormone signaling, and osmolyte synthesis [7,8,9]. Anatomical and morphological changes in response to drought stress include the reduction in leaf number and leaf area to reduce water loss and the increase in root growth for enhanced water uptake, whereas, in heat stress, the increased transpiration is achieved by increasing leaf number and area [10].

However, much remains to be discovered about the underlying mechanisms of systemic plant response to combinations of stress [10,11,12]. In such an endeavor, an integrated systems biology approach would potentially shed light on the gene function towards a better understanding of the abiotic stress response mechanisms for developing climate-change-oriented breeding strategies.

A wide spectrum of abiotic stresses, either single or combined, can impair plant function by inhibiting essential processes and induce overproduction of ROS, which are extremely reactive and toxic, causing damage to proteins, lipids, carbohydrates and DNA and ultimately resulting in oxidative stress [13]. Antioxidant mechanisms play an important role in the response of plants to a combination of drought and heat stress, with higher antioxidant capacity being associated with plant tolerance against stress combination [6,14]. Glutathione-S-transferases (GST, EC 2.5.1.18.) are members of the enzymatic defense system exhibiting high divergence with multiple functions in stress-tolerance [15], and their expression is tissue and/or developmental stage-specific and differs in response to stresses [16]. Especially members of the Tau and Phi classes have been shown to mitigate the effects of environmental stress factors, such as water deficit stress [17], salinity [18], heavy metal toxicity [19,20] and pathogen attack [21].

Several GST, both constitutive and induced, are expressed in plants, and their expression in different plant tissues (leaves and roots) is dependent on the sensed stimuli [22]. Thus, far, studies regarding the effect of combined or single drought and heat stresses reinforce the evidence of the significant role of antioxidant enzymes in a combination of drought and heat stress. For instance, the cytosolic ascorbate peroxidase 1 (APX1) has been found to play a key role in the acclimation of plants to a combination of drought and heat stress [23]. Even more, the protection of the antioxidative enzymes, such as the superoxide dismutase (SOD) and guaiacol peroxidase (GPX) involved in the control of ROS in stress conditions has been found to reduce the negative effects of heat, drought and light stresses and their combinations [24]. Increased GST activity has been observed under high temperature and/or drought stress, compared to control mung bean seedlings [25], or was induced under combined and single drought and heat stresses in an abscisic acid (ABA)-independent manner, yet when combined stress occurred, the effect on GST expression was interactive [26]. Recent studies on tobacco plants overexpressing the *Gmgstu*4 suggest pleiotropic effects on plant metabolism under salt stress, where the transgenic plants exhibited an increased concentration of protective metabolites, such as proline and trehalose [27], little is known about the regulatory functions of GSTs in combined stress plant response.

*Phaseolus vulgaris* (common bean) is a major grain legume crop and is among the five cultivated species from the genus Phaseolus with great economic and commercial importance after soybean and peanut [28]. The common bean crop is cultivated in diverse environments, such as drought-prone areas, and studies have shown that it is sensitive to many abiotic stresses, including water limitation [29]. Both drought and salinity stresses have a negative impact on the common bean crop production, especially when the stress occurs in specific growth stages of the crop [30]. However, there is some variability within *P. vulgaris* for salt and drought-tolerance [31,32]. Herein, the stress-induced *Pvgstu* [21,33] was isolated from a Greek drought-tolerant variety “Plake Μegalosperma Prespon” of protected geographic indication (PGI) (reg. 18.07.1998) [34,35] cultivated in the areas around Prespa lakes, North West Greece and its mature white seeds are harvested as dry beans. GSTs are major stress regulators playing an important role in the tolerance mechanism involved under abiotic stresses. The transgenic-based approaches have been, thus far, applied to enable the understanding of mechanisms against mainly single abiotic stress damage for the development of tolerant plant species and varieties [36]. The aim of this study was to better understand the functional role of the *Pvgstu* as a target gene in plant stress response against heat, water deficit and combined heat and drought stresses via overexpression into the model plant tobacco. Our hypothesis is that abiotic- or herbicide-inducible *gstu* genes would potentially confer systemic protection against abiotic stresses and was relied on preliminary data indicating that the *Pvgstu* transgenic plants exhibited an enhanced performance under a series of single abiotic and oxidative stresses (unpublished results). More specifically, we have used a biotic-stress -inducible (*Uromyces appendiculatus—*responsible for bean rust) glutathione transferase isolated from the roots (*Pvgstu3–3*) and a herbicide (fluazifop-p-butyl)-inducible GST isolated from the leaves (*Pvgstu2–2*) of *P. vulgaris* PGI variety “Plake Μegalosperma Prespon” for their high antioxidant catalytic function and action as hydroperoxides, thioltransferases, and dehydroascorbate reductases [21,33]. By overexpressing each of these genes in the model plant, *N. tabacum*, we aimed at unraveling whether and how such GSTs derived from different plant tissues of the common bean may affect the response to drought, high temperature and their combination. To our knowledge, the role of *gstu* genes from the common bean (*P. vulgaris*) has not yet been investigated. Unravelling the metabolomic and transcriptomic aspects of plant response to the combination of heat and drought will elucidate the pathways involved in the in planta potential of *Pv*GSTU isoenzymes to mitigate the negative impacts of combined abiotic stresses in stress-prone areas and especially under the current predictions of global climate change.

## 2. Results

### 2.1. Evaluation, Relative Expression and Enzymatic Activity of the Transgenes

Double digestion and ligation were performed to generate the recombinant plasmids pART27-*Pvgstu3–3* and pART27-*Pvgstu2–2* under the control of the CaMV35S promoter and were verified by PCR (Figure S1a). The recombinant plasmids were transferred to *E. coli,* and the colonies were screened for successful insertion of the plasmid (Figure S1b,c) (More details can be found in the supplementary material). The positive colonies were further tested by restriction enzyme analysis and were sequenced.

For the genetic transformation of the leaf explants, an initial co-cultivation with the *A. tumefaciens* was performed, followed by four weeks in the selection medium MSR1. In total, 21 and 10 putative genetically transformed independent T0 lines overexpressing the *Pvgstu2–2* and *Pvgstu3–3* genes, respectively, with resistance to kanamycin, were obtained. RT–PCR analysis verified that 19 out of 21 putative transgenic lines were positive for the *Pvgstu2–2* (~90.5% success) and 9 out of 10 (~90% success) for the *Pvgstu3–3*. The expected 853 bp and 822 bp fragments were detected in the independently transformed tobacco lines for the *Pvgstu2–2* and *Pvgstu3–3,* respectively, whereas no band was observed in the wild-type (WT) plants (Figure S2).

Quantitative RTPCR revealed varied transcript levels among the transgenic lines as compared to the non-detectable expression in WT plants. More specifically, in comparison to the *β-actin* expression levels, the *Pvgstu2–2* gene showed higher levels of expression in lines *Pvgstu2–2.9* (1.99 ± 0.33), *Pvgstu2–2.16* (1.00 ± 0.04), *Pvgstu2–2.19* (0.99 ± 0.25) and *Pvgstu2–2.13* (0.93 ± 0.55). The *Pvgstu3–3* gene was highly expressed in the T0 *Pvgstu3–3.4* (77.71 ± 0.51), *Pvgstu3–3.10* (51.62 ± 0.47), *Pvgstu3–3.2* (38.05 ± 0.40), *Pvgstu3–3.8* (22.63 ± 0.61) and *Pvgstu3–3.28* (20.11 ± 0.74) lines (Figure S3; Table S1).

For the enzymatic activity, eight lines overexpressing the *Pvgstu2–2* gene were tested on the NBD-CI substrate, with seven lines showing similar enzyme activity and *Pvgstu2–2.13* line having 2-times higher enzymatic activity compared to the rest. Respectively, from the nine lines overexpressing the *Pvgstu3–3* gene, the *Pvgstu3–3.1*, *Pvgstu3–3.4* and *Pvgstu3–3.8* showed the highest enzymatic activity compared to non-genetically transformed plants in CDNB substrate (Figure S4). These results confirmed that the T0 transgenic lines encode for active GST proteins.

### 2.2. Morpho-Physiological Responses

The T1 independent transgenic tobacco lines *Pvgstu2–2.19* and *Pvgstu3–3.4* were selected for this study based on two factors: (i) the high relative expression and enzymatic activity of the transgenes (Figures S3 and S4; Table S1) and (ii) their previous performance under salinity and drought stresses (unpublished results as part of the GST-TOOLS research project, THALES Program). Therefore, to further assess the in planta role in abiotic stress response mechanisms of the two *Pvgstu* genes isolated from the roots and the leaves of *P. vulgaris*, we assessed the morphophysiological, transcriptomic and metabolomic response of WT and transgenic tobacco lines *Pvgstu2–2.19* and *Pvgstu3–3.4* in vivo after applying combined (DT) and single drought (D) and heat (T) stresses in growth chamber-controlled conditions.

**Figure 1:**
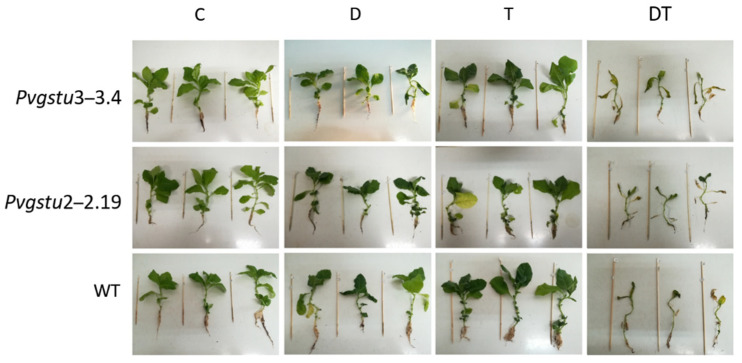
Effect of heat (T), drought (D) and combination of drought and heat (DT) stress on the growth of transgenic lines (*Pvgstu3–3.4* and *Pvgstu2–2.19*) and wild-type (WT) tobacco plants after 9 days compared to the stress-free, control (C) conditions. It is interesting that under single D or T stresses did not show any significant wilting as compared to the DT treatment.

**Figure 2:**
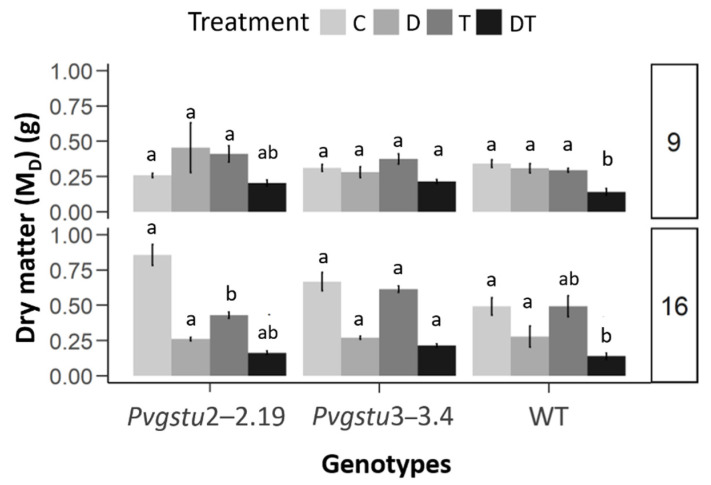
Dry matter (M_D_) (g) of the transgenic lines *Pvgstu3–3.4* and *Pvgstu2–2.19* and WT plants growing under control (C), drought (D), heat (T) and combination of drought and heat stresses (DT) at nine and 16 days. Different letters indicate significant differences among genotypes for each treatment at *p* < 0.05. Data are means ± SE (WT: *n* = 4; *Pvgstu* lines at 9 days: *n* = 5; at 16 days: *n* = 7).

To investigate the specific effects of the single drought and heat stresses and their combinatorial effect on growth parameters, two harvests were performed: at nine days (mid-harvest) and 16 days (final harvest). Additionally, the transgenic lines and WT plants under DT stress showed significant leaf wilting from day nine onward. Therefore, re-watering was applied on day 9 to half of the DT-treated plants to assess whether the plants would effectively recover in comparison to the DT plants on day 16 (final harvest). The plants remained under heat to test whether the wilting was attributed to drought or heat stress, given that plants under single D or T stresses did not show any significant wilting. Overall, we observed that (i) the combined stress treatment was more severe compared to single stress effects, as early as on day six (Figure S5) and (ii) the *Pvgstu3–3.4* was more tolerant compared to *Pvgstu2–2.19* and WT plants. It is interesting that under single D or T stress, neither the transgenic lines nor the WT plants showed any significant wilting compared to the DT treatment as observed on mid-harvest (day 9) (Figure 1). This was consistent with maintaining their dry weight under single stress treatments (Figure 2), yet under DT stress, the *Pvgstu3–3.4* dry weight was significantly higher compared to WT (Figure 2). The *Pvgstu3–3.4* was generally more tolerant to the stresses compared to WT plants and *Pvgstu2–2.19*, with the latter showing a more intermediate response (Table 1). Specifically, under D and DT treatments, both the *Pvgstu3–3.4* and WT had a similar response trend in terms of fresh weight and leaf number (Figure 2; Table 1). However, the major difference was observed in root length, with *Pvgstu3–3.4* being unaffected even under severe combined DT stress, whereas the *Pvgstu2–2.19* and WT plants showed a moderate reduction under the single stresses (T, D) and a 54.4 and 53.8% reduction under combined DT stress and C conditions, respectively (Table 1).

Based on the final harvest data (day 16), *Pvgstu3–3.4* outperformed the *Pvgstu2–2.19* and WT plants under single T stress in all morphological parameters (Table 2; Table S2), with greater root length, fresh- and dry weights and by showing significantly greater dry weight in combined DT stress and fresh weight at recovery (Figure 2; Table 2). Nevertheless, the utmost advantage of the *Pvgstu2–2.19* line was the significantly higher leaf number not only under control conditions but also under severe combined DT stress when compared to WT plants (Table 2). Single D treatment did not have any negative effect on the morphological parameters measured, however under combined DT stress, the root length was significantly reduced in *Pvgstu2–2.19*, whereas the WT plants showed significant leaf loss (Table 2) and a significant reduction in dry matter (M_D_) (Figure 2). The recovery of both transgenic lines and WT plants previously subjected to DT treatment was successful, as observed on the final harvest (day 16), with *Pvgstu3–3.4* exhibiting increased turgor manifested with significantly increased M_F_ (Table 2). M_F_ was improved at recovery to a greater extent than plants under single D or combined DT treatments, and leaf loss was more severe under DT stress in all transgenic lines and WT plants (Table S2).

**Figure 3:**
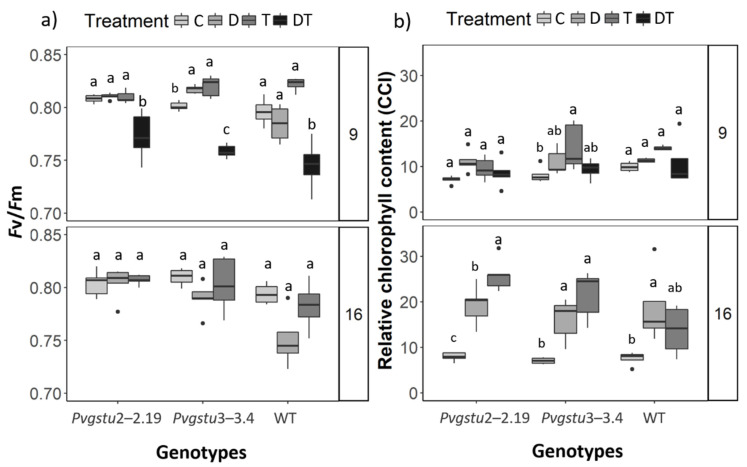
Physiological measurements: (**a**) maximum fluorescence (*F*v/*F*m) of photosystem II and (**b**) relative chlorophyll content (CCI) measured in the transgenic lines *Pvgstu3–3.4* and *Pvgstu2–2.19* and WT plants growing under control (C), drought (D), heat (T) and combination of drought and heat stresses (DT), at nine and 16 days. Different letters indicate significant differences among treatments for each genotype at *p* < 0.05. Data are means ± SE (WT: *n* = 4; *Pvgstu* lines at 9 days: *n* = 5; at 16 days: *n* = 7).

The combination of DT stress negatively affected the functionality of PSII in both transgenic and WT plants with a dramatic decrease in maximum quantum yield of PSII on day 9 (mid-harvest), while during the final harvest (day 16), we could not obtain any data due to the extensive leaf wilting and senescence (Figure 3). Nevertheless, both transgenic lines showed a greater maximum quantum yield of PSII under single D stress on days 9 and 16 compared to the WT plants (Figure 3; Table S3). At nine days, the relative chlorophyll content increased in *Pvgstu3–3.4* under single T stress, whereas it was not affected under any stress treatment in *Pvgstu2–2.19* and WT plants compared to stress-free plants. However, at 16 days, *Pvgstu3–3.4* increased the relative chlorophyll content under single D and T stresses, while *Pvgstu2–2.19* only under single T stress and the WT plants under D stress compared to the stress-free control plants (Figure 3; Table S3). In the combined DT stress, the leaves of all transgenic lines and WT plants senesced (Figure 3); yet, the recovery of the physiological parameters on day 16, after seven days from the beginning of re-watering (day 9), was significant for all transgenic and WT plants compared to the DT plants on day 9, given that no physiological data could be obtained from DT treatment at 16 days (Table S4).

### 2.3. Effect of Pvgstu3–3 Overexpression on the Transcriptome in Control and Combined Drought and Heat Stress Conditions

Based on the morphophysiological results, the *Pvgstu3–3.4* line showed better response under a combination of heat and drought stresses, and therefore, it was selected for further investigation of the alterations in transcriptome and metabolome levels to understand the potential response of a biotic-induced gene under concurrent heat and drought stress. The comparison was performed among the WT plants under control conditions (group 1) and transgenic line *Pvgstu3–3.4* under control (group 2) and 8 h (group 3) and 48 h (group 4) after exposure to combined DT with an average of 75.64% reads being mapped to the reference genome. The uniformity of the mapping result for each sample reinforced that the expression profile was similar, thus allowing for their comparative analysis. The analysis of all 4 groups generated 42,538 million clean reads in total with a quality score Q20 (error probability of 1%) at 98.27%. In terms of gene expression, the total mapping rate was on average 83.79%, with a unique mapping rate being at 20.24%. The total number of identified genes was, on average, 77.5 million, of which 49.2 million are known genes.

**Figure 4:**
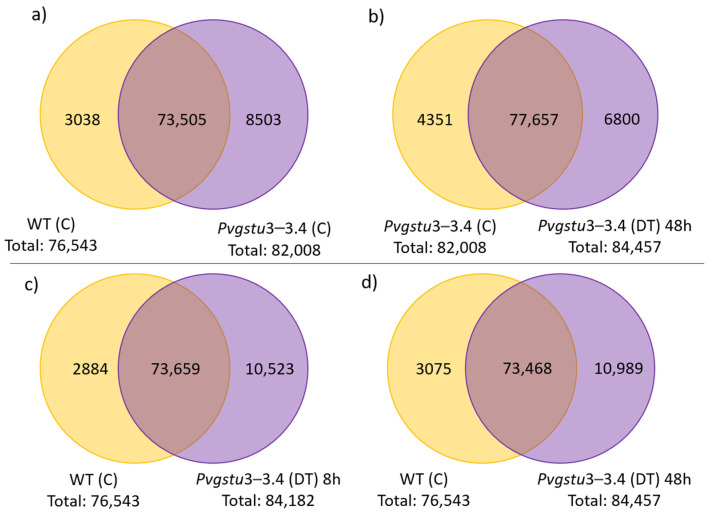
Venn diagram of differentially expressed genes (DEGs). Comparison between (**a**) WT vs. *Pvgstu3–3.4* in stress-free control (C) conditions (groups 1 vs. 2, respectively); (**b**) stress-free *Pvgstu3–3.4* (C) vs. *Pvgstu3–3.4* at 48 h under DT (groups 2 vs. 4, respectively); (**c**) stress-free WT (C) vs. *Pvgstu3–3.4* at 8 h under DT (groups 1 vs. 3, respectively); and (**d**) stress-free WT (C) vs. *Pvgstu3–3.4* under 48 h DT (groups 1 vs. 4, respectively). The intersection of the two circles represents overlapping DEGs between the representative datasets.

The differentially expressed genes (DEGs) were selected according to the standard of *p*< 0.05, and the false discovery rate (FDR) was set to 0.001 to determine the threshold of the *p*-value for multiple tests. The absolute value of |log2Ratio| ≥ 1 was used to determine the difference between the gene expression transcription group and the database. Analysis of the DEGs between the stress-free (control conditions) WT and transgenic plants (group 1 vs. group 2) showed that there are 73,505 commonly expressed DEGs, 8503 unique DEGs expressed in *Pvgstu3–3.4* and 3038 in WT (Figure 4a). However, when the combined DT stress was applied, we observed that *Pvgstu3–3.4* under control conditions compared to 48 h under combined DT stress (group 2 vs. group 4) had more commonly expressed DEGs (77,657), 4351 unique DEGs for *Pvgstu3–3.4* in stress-free conditions and 6800 for *Pvgstu3–3.4* at 48 h under DT stress (Figure 4b). A slight increase in the uniquely expressed DEGs (10,989) was observed in *Pvgstu3–3.4* at 48 h under DT stress (Figure 4d) compared to 10,523 after 8 h under DT stress (Figure 4c), which were greater than the uniquely expressed DEGs of stress-free *Pvgstu3–3.4* (Figure 4a). After comparing the commonly expressed genes between stress-free conditions (Figure 4a), 8 h under DT stress (Figure 4c) and 48 h under DT stress (Figure 4d), we observed an increase of 154 commonly expressed DEGs at 8 h (Figure 4c) and a decrease of 37 commonly expressed DEGs at 48 h under DT stress (Figure 4c) compared to the stress-free conditions (Figure 4a).

**Figure 5:**
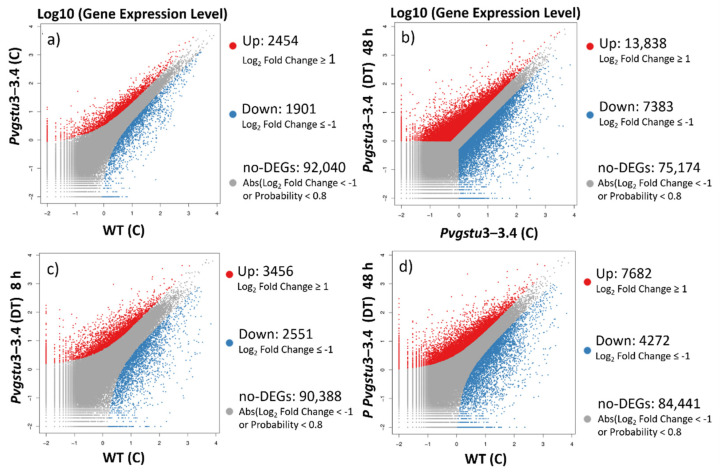
Scatter plot depicting the correlation of the gene expression profiles between (**a**) WT vs. *Pvgstu3–3.4* in stress-free control (C) conditions (0 h; groups 1 vs. 2, respectively); (**b**) stress-free *Pvgstu3–3.4* (C) vs. *Pvgstu3–3.4* at 48 h under DT (groups 2 vs. 4, respectively); (**c**) stress-free WT (C) vs. *Pvgstu3–3.4* at 8 h under DT (groups 1 vs. 3, respectively).; (**d**) stress-free WT (C) vs. *Pvgstu3–3.4* at 48 h under DT (groups 1 vs. 4, respectively). DT indicates combined drought and heat stresses. The significantly up- and downregulated genes with probability ≥ and ≤ 0.8, respectively, are presented with red and blue dots, respectively, while non-significant genes as gray dots.

A total of 4355 unigenes were identified between WT and *Pvgstu3–3.4* (groups 1 against 2, respectively) under stress-free conditions, including 2454 up- and 1901 downregulated genes (Figure 5a). However, when we compared the *Pvgstu3–3.4* under C conditions against combined DT stress at 48 h, a total of 21,221 unigenes (~5-fold greater than stress-free *Pvgstu3–3.4* against WT) were identified with 13,838 up- and 7383 downregulated genes (Figure 5b). Between *Pvgstu3–3.4* at 8 h (Figure 5c) and 48 h (Figure 5d) under DT stress, the identified unigenes increased by 2-fold with a total of 6007 and 11,954 unigenes, respectively.

Gene Ontology (GO) assignments (biological process, cellular component and molecular function) were used to classify the predicted functions of WT and *Pvgstu3–3.4* line under control and DT conditions at the different time points (Figure S6). Between stress-free WT (C) and *Pvgstu3–3.4* under C (Figure S6a) and DT conditions at 8 h (Figure S6c) and 48 h (Figure S6d), we observed a progressive increase in the DEGs. Overall, similar GO assignments were represented among the WT against *Pvgstu3–3.4* in stress-free conditions (Figure S6a) and *Pvgstu3–3.4* under DT conditions at 8 h (Figure S6c) and 48 h (Figure S6d), yet the associated DEGs increased with the longer exposure to DT stress (8 h against 48 h). Among the biological processes, DEGs involved in the nitrogen utilization, rhythmic processes and pigmentation were induced under DT stress at both 8 h (Figure S6c) and 48 h (Figure S6d) when compared to the control conditions (Figure S6a). In addition, cell aggregation, cell proliferation, detoxification and cell junction were induced after 48 h of DT imposition. Interestingly, the *Pvgstu3–3.4* DT against control conditions (Figure S6b) exhibited a higher number of DEGs for all GO terms.

The DEGs were assigned by the KEGG according to functional classification and biochemical pathways, as shown in Table 3 for the WT and *Pvgstu3–3.4* under stress-free and DT conditions in the different time points (8 and 48 h). KEGG pathway analysis revealed that between the *Pvgstu3–3.4* and the WT plants under stress-free conditions, the representative pathways included: the metabolic pathways (24.56%), mitogen-activated protein kinase (MAPK) signaling pathway (6.01%) and NF-kappa B signaling pathway (5.48%). Additionally, 2.47% of the DEGs were assigned to the arachidonic acid metabolism, tryptophan metabolism and peroxisome pathways (Table 3). However, when comparing the WT under stress-free conditions against the *Pvgstu3–3.4* at 8 h under DT, the metabolic pathways were at a slightly increased percentage (26.05%) and included other pathways, such as the starch and sucrose metabolism (2.99%), amino sugar and nucleotide sugar metabolism (2.69%), the galactose metabolism, arachidonic acid metabolism, retinol metabolism at 2.10% and the metabolism of xenobiotics by cytochrome P450 and tryptophan metabolism at 1.95% (Table 3). At 48 h under DT, the represented pathways aside from metabolic pathways (24.87%) included the carbon metabolism (3.46%), biosynthesis of amino acids (2.52%), starch and sucrose metabolism (2.46%), tryptophan metabolism (1.76%), arachidonic acid metabolism (1.70%), amino sugar and nucleotide sugar metabolism, retinol metabolism at 1.64%, whereas metabolism of xenobiotics by cytochrome P450, glycolysis/gluconeogenesis and glyoxylate and dicarboxylate metabolism at 1.51% (Table 3). Interestingly, when comparing *Pvgstu3–3.4* under DT at 48 h in reference to control conditions, DEGs of carbon metabolism (3.43%), AMPK signaling pathway, biosynthesis of amino acids and ABC transporters at 2.17%, pyruvate metabolism (1.79%), starch and sucrose metabolism (1.17%) and citrate cycle (TCA cycle) (1.39%) were involved in response to combined drought and heat stresses (Table 3).

In stress-free conditions (Table 3 and Table S7a), the genes coding cytochrome P450 families 1 and 2 involved in the metabolism of xenobiotics by cytochrome P450 and tryptophan metabolism were upregulated in the transgenic plants overexpressing *Pvgstu3–3.4* (group 2) as compared to WT (group 1), along with the genes for glucuronosyltransferase involved in both ascorbate and aldarate metabolism, retinol metabolism, porphyrin and chlorophyll metabolism, metabolism of xenobiotics by cytochrome P450 and pentose-glucuronate interconversions. Increased transcript levels coding MAPK, an important signal transduction cascade that responds to an external signal, were shown in the stress-free transgenic *Pvgstu3–3.4* plants. More specifically, the dual-specificity MAP kinase phosphatase (MKP), serine/threonine-protein phosphatase 2B catalytic subunit (PPP3C) and protein phosphatase 1B (PP2CB) were upregulated, whereas the mitogen-activated protein kinase 4 (MEKK4) and MEKK2/3 were downregulated. Interestingly, 31 genes involved in the NF-kappa B signaling pathway were differentially expressed in the transgenic plants, with the ubiquitin-conjugating enzyme E2 I (UBC9) BGI_novel_G037382 gene (2.8-fold) (Table S7a) and the interleukin-1 receptor-associated kinase 1/4 (EC:2.7.11.1) coding genes being upregulated even under stress-free conditions. In the arachidonic acid metabolism, the downregulated genes in *Pvgstu3–3.4* included those encoding for arachidonate 5-lipoxygenase (EC:1.13.11.34) and arachidonate 15-lipoxygenase (EC:1.13.11.33), whereas leukotriene-B4 20-monooxygenase related genes were upregulated. Gene expression alterations were detected in the linoleic acid metabolism and, more specifically, in the genes encoding for linoleate 9S-lipoxygenase (EC:1.13.11.58), which is also involved in lipid metabolism and was found to be downregulated. In the tryptophan metabolism, the genes encoding for L-amino-acid oxidase (EC:1.4.3.2) and enoyl-CoA hydratase (EC:4.2.1.17) were also downregulated, suggesting a possible reduction in acetyl CoA and, therefore, reduction in the glycolysis rate. In the amino sugar and nucleotide sugar metabolism pathway, the chitinase and N-acetylglucosamine kinase involved in the synthesis of UDP-sugars, which are considered important for biomass production, were upregulated. In the same pathway, genes for the reversibly glycosylated polypeptide, UDP-arabinose 4-epimerase, UDP-glucose 4-epimerase, xylan 1,4-beta-xylosidase and alpha-1,4-galacturonosyltransferase were downregulated. The peroxisome xanthine dehydrogenase/oxidase in purine metabolism, the dehydrogenase/reductase SDR family member 4 in the retinol metabolism along with superoxide dismutase (SOD), Cu-Zn family (EC:1.15.1.1) in the antioxidant system were upregulated. In phenylalanine metabolism, the L-amino-acid oxidase (EC:1.4.3.2) genes also involved in other modules of the amino acid metabolism were downregulated, mainly leading to reduced phenylpyruvate or increased tyrosine through the conversion of phenylalanine. Yet, the 4-coumarate--CoA ligase (EC:6.2.1.12), caffeoyl-CoA O-methyltransferase (EC:2.1.1.104) and cytoplasmic aspartate aminotransferase (EC:2.6.1.1) were upregulated.

Between stress-free WT (group 1) and *Pvgstu3–3.4* at 48 h under DT (group 4) (Table 3 and Table S7c), we observed that glutathione S-transferase (EC:2.5.1.18) and glucuronosyltransferase (EC:2.4.1.17) genes were upregulated in the metabolism of xenobiotics by cytochrome P450 pathway. Furthermore, in the starch and sucrose metabolism, the sucrose-phosphate synthase (EC:2.4.1.14), beta-glucosidase (EC:3.2.1.21), isoamylase (EC:3.2.1.68) and alpha-trehalase (EC:3.2.1.28) were upregulated along with alpha-amylase (EC:3.2.1.1) (increased maltose) and maltooligosyl trehalose trehalohydrolase (EC:3.2.1.141), whereas only glycogen phosphorylase and trehalose 6-phosphate synthase were downregulated. Moreover, in the glycolysis/gluconeogenesis pathway, the glucose-6-phosphate 1-epimerase, fructose-1,6-bisphosphatase I and 6-phosphofructokinase 1 were upregulated, indicating a possible increase in pyruvate and along with the upregulated pyruvate dehydrogenase E1 component, acetyl-CoA synthetase and isocitrate dehydrogenase (NAD ^+^ ) in the TCA cycle, they may contribute to the protective role against oxidative stress. It is also interesting that for the hexokinase fructose-bisphosphate aldolase, both up- (BGI_novel_G016405 (3.1-fold)) and downregulated (BGI_novel_G016404 (−2.9-fold), BGI_novel_G016402 (−1.9-fold)) (Table S7c) genes were observed, indicating possible perturbations in the production of fructose in *Pvgstu3–3.4* after 48 h under combined DT stress compared to the WT plants. Regarding the tryptophan metabolism pathway, amidase was also involved in the arginine and proline metabolism, and phenylalanine metabolism was upregulated. Additionally, genes encoding for acetyl-CoA C-acetyltransferase (EC:2.3.1.9), also implicated in other pathways, such as fatty acid degradation, valine, leucine and isoleucine degradation, lysine degradation and tryptophan metabolism, were downregulated. Interestingly, shikimate kinase (EC:2.7.1.71) was upregulated along with the anthranilate phosphoribosyltransferase (EC:2.4.2.18) and indole-3-glycerol phosphate (IGP) [4.1.1.48] in the tryptophan biosynthetic pathway, indicating a potential role in the tryptophan-independent IAA de novo biosynthetic pathway. Several genes in the AMPK signaling pathway encoding for the 5′-AMP-activated protein kinase, catalytic alpha subunit (EC:2.7.11.11) were upregulated as a result of the upregulated genes of calcium/calmodulin-dependent protein kinase 2 (EC:2.7.11.17), serine/threonine-protein phosphatase 2A catalytic subunit (EC:3.1.3.16), acetyl-CoA carboxylase/biotin carboxylase 1 and MFS transporter (facilitated glucose transporter). The increased regulation of these genes possibly led to the upregulation of fructose-1,6-bisphosphatase I (EC:3.1.3.11), 6-phosphofructokinase 1 (PFK1) (EC:2.7.1.11) and 6-phosphofructo-2-kinase/fructose-2,6-biphosphatase 1 (EC:2.7.1.105/3.1.3.46], which are related to the glycolysis/gluconeogenesis pathway. Notably, genes related to the biosynthesis of amino acids, such as proline biosynthesis in arginine and proline metabolism, valine, leucine and isoleucine biosynthesis, cysteine and methionine metabolism and histidine metabolism were induced in response to DT treatment at 48 h in the *Pvgstu3–3.4* plants.

To further understand the combined DT effect on the transcriptome of the *Pvgstu3–3.4* transgenic line, we compared the transgenic control plants (group 2) against the transgenic plants under combined DT (group 4), which revealed significant alterations in the regulation of several genes involved in different metabolic pathways (Table 3 and Table S7d). Among the metabolic pathways, carbon metabolism was largely enriched under DT conditions in *Pvgstu3–3.4* plants. Genes coding 6-phosphofructokinase 1 (EC:2.7.1.11), fructose-1,6-bisphosphatase I (EC:3.1.3.11), phosphoribulokinase (EC:2.7.1.19), ribose 5-phosphate isomerase A (EC:5.3.1.6), ribulose-phosphate 3-epimerase (EC:5.1.3.1) being involved in pentose phosphate cycle and Calvin cycle were upregulated. Additionally, genes coding pyruvate kinase (EC:2.7.1.40), phosphoglycerate kinase (EC:2.7.2.3) and glyceraldehyde 3-phosphate dehydrogenase (EC:1.2.1.12) involved in glycolysis (Embden–Meyerhof pathway) for the conversion of glucose to pyruvate were also upregulated. However, the hexokinase (EC:2.7.1.3) and fructose-bisphosphate aldolase, class I (EC:4.1.2.13) involved genes were downregulated, and fructose-1,6-bisphosphatase I (FBP) (EC:3.1.3.11), fructose-2,6-bisphosphatase (EC:3.1.3.46), GDP-L-fucose synthase (EC:1.1.1.271) and fucokinase (EC:2.7.1.52) were upregulated. Another important metabolic pathway implicated in stress-tolerance is starch and sucrose metabolism, in which we observed that the genes encoding for maltase-glucoamylase were upregulated (participating in fructose biosynthesis), as well as those of beta-glucosidase and maltase-glucoamylase, leading to glucose biosynthesis and alpha-amylase leading to enhanced maltose biosynthesis. In the glycolysis/gluconeogenesis pathway, genes coding pyruvate oxidation, such as aldehyde dehydrogenase (NAD(P) ^+^ ) (EC:1.2.1.5), among others, were also mainly upregulated. In addition, the upregulation of pyruvate kinase implicated not only in glycolysis/gluconeogenesis but also in pyruvate metabolism may lead to increased pyruvate. In parallel, the upregulation of glycerate dehydrogenase and hydroxypyruvate reductase in glycine, serine and threonine metabolism could also enhance pyruvate production. At the same pathway, the gene encoding tryptophan synthase alpha chain, along with genes in the tryptophan metabolism, were upregulated, leading to tryptophan production.

Biosynthesis of amino acids was also strongly induced under DT stress; the glutamate family pathway was upregulated under stress, such as the glutamate 5-kinase and glutamate-5-semialdehyde dehydrogenase related gene BGI_novel_G018845 (2.1-fold increase) and pyrroline-5-carboxylate reductase BGI_novel_G038528 gene (1.3-fold increase), leading to the accumulation of the compatible osmolyte, proline (Table S7d). Upregulation of the pyruvate family pathway genes (BGI_novel_G023627 (2.9-fold increase), BGI_novel_G004872 (1.1-fold increase)) and enhanced production and accumulation of alanine, and the branched-chain amino acids, isoleucine, leucine and valine (BGI_novel_G019491 (6.9-fold), BGI_novel_G019481 (4.4-fold), BGI_novel_G002984 (3.5-fold), BGI_novel_G019480 (3.1-fold), BGI_novel_G002987 (3.0-fold), BGI_novel_G018935 (2.9-fold), BGI_novel_G020821 (2.5-fold), BGI_novel_G014739 (1.8-fold) and BGI_novel_G025500 (2.4-fold)) (Table S7d), which under stress function as compatible solutes may provide an alternative source of respiratory substrates. However, genes related to the aspartate family pathway, essential for generating energy mainly via lysine catabolism, appeared to be either upregulated, such as BGI_novel_G012015 (3.6-fold) BGI_novel_G007764 (1.8-fold) and BGI_novel_G029903 (1.5-fold) or downregulated, such as BGI_novel_G007765 (−1.2-fold) and BGI_novel_G025320 (−3.6-fold) (Table S7d). In the arginine biosynthesis and alanine, aspartate and glutamate metabolism pathways, the glutamine synthetase (EC:6.3.1.2), alanine transaminase (EC:2.6.1.2) and cytoplasmic aspartate aminotransferase (EC:2.6.1.1) related transcripts were upregulated, leading to an increase in arginine and alanine, which may lead to fumarate involved in the citrate cycle (TCA cycle).

This was confirmed by the induction of TCA cycle metabolism-related genes involved in the second carbon oxidation cycle, methylaspartate cycle and pyruvate oxidation which were upregulated. In the photorespiration metabolism gene expression was upregulated between 7.8- and 1-fold (BGI_novel_G002321 (7.8-fold), BGI_novel_G004953 (5.3) coding for glycine dehydrogenase (EC:1.4.4.2), BGI_novel_G035362 (3.4-fold), BGI_novel_G006553 (2.8-fold), BGI_novel_G035361 (2.6-fold), BGI_novel_G001601 (2.1-fold) and BGI_novel_G028763 (1.1-fold)) (Table S7d) compared to the control conditions. It is also worth mentioning that the genes involved in the generation of acetyl coenzyme A (acetyl-CoA) pools were also upregulated under DT conditions. The endocytosis mechanism required for nutrient uptake and signaling transduction was largely affected under DT conditions, with a large number of genes being upregulated. ATP-binding cassette (ABC) transporters were mainly upregulated. In addition, genes encoding for antioxidant enzymes, such as superoxide dismutase (SOD) (BGI_novel_G028763 (1.1-fold)) and Catalase (CAT) (BGI_novel_G028808 (2.7-fold), BGI_novel_G009557 (1.8-fold), BGI_novel_G029941 (1.3-fold) and BGI_novel_G004146 (1.3-fold)) (Table S7d) were overexpressed in the peroxisome. In the linoleic acid pathway, the BGI_novel_G023971 gene encoding secretory phospholipase A2 (EC:3.1.1.4), which is involved in lecithin production, was 2.6-fold upregulated in the DT combined stress treatment (Table S7d). In the metabolism of xenobiotics by cytochrome P450, glutathione S-transferase (GST) (EC:2.5.1.18), aldehyde dehydrogenase (NAD(P) ^+^ ) (EC:1.2.1.5) and glucuronosyltransferase (EC:2.4.1.17) and carbonyl reductase 1 (EC:1.1.1.184) were upregulated at 48 h under combined DT stresses.

The DEGs involved in transcriptional regulation were surveyed to investigate the role of *Pvgstu3–3.4* overexpression in response to combined DT stress. Major transcription factors involved the NAC, MYB, ERF, bZIP, WRKY and HSP types (Table 4). Among the DEGs, a total of 2918 TFs grouped into 56 families were identified. In total, 218 were upregulated, and 210 downregulated in *Pvgstu3–3.4* under control conditions compared to the WT plants. At 48 h under DT stress, the TFs in *Pvgstu3–3.4* increased to 463 up- and 445 downregulated, compared to WT under control conditions. However, we observed a higher activity in the TFs of *Pvgstu3–3.4* under DT conditions against stress-free conditions (816 up- and 766 downregulated). In general, the number of TFs increased in accordance with the length of exposure to DT conditions (48 h >8 h under DT > stress-free conditions). The most abundant and active TFs were the MYB, mTERF, MYB-related, AP2-EREBP, and NAC families (Table 4). However, other stress-related TFs were among the top 15 TF families, such as the FAR1, MADS, AP2-EREBP, NAC, Trihelix, ARF, HSF, ARR-B, WRKY, bZIP, SBP and Sigma70-like (Table 4). Interestingly, DEGs from the MYB, MYB-related, AP2-EREBP, NAC, HSF and WRKY TF families were expressed even under nonstress conditions and were increased in *Pvgstu3–3.4* plants after exposure to a combination of DT stress.

### 2.4. Changes in the Metabolome

**Figure 6:**
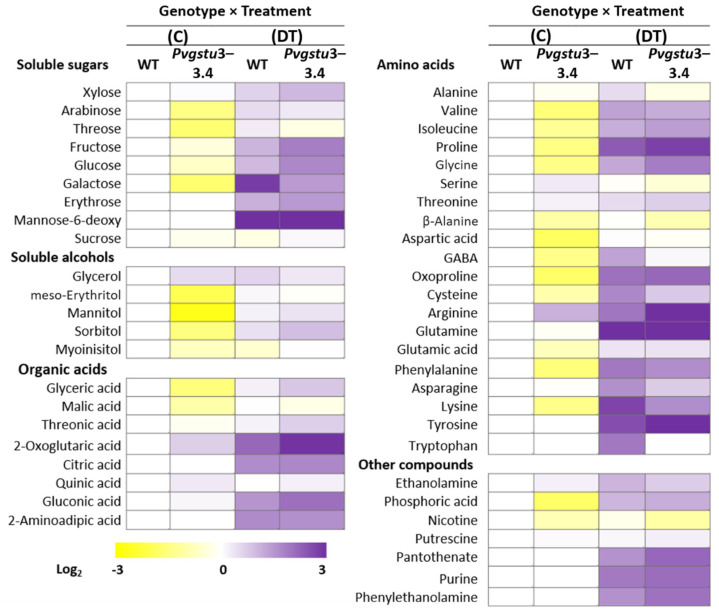
Heat map of primary metabolites of *Pvgstu3–3.4* and WT plants under combined DT stress (9 days) compared to control conditions. For each metabolite, the ratio between DT treatment to control was transformed into log2 and depicted with a color scale indicated as purple (increase) and as yellow (decrease; see color scale). Mean values of three independent determinations for each treatment were expressed as relative abundance compared to internal standard adonitol and are reported as background-free of the control conditions at 0 days. Actual data are provided in Supplementary Table S6.

The increased stress-tolerance of *Pvgstu3–3.4* to drought and high temperatures was investigated further through the metabolic changes under control and combined stress conditions on day nine for investigating stress response and plant acclimation under these conditions. Plants overexpressing the *Pvgstu3–3.4* upregulated the central metabolism, as levels of sugars, amino acids and TCA cycle intermediates were in general increased. We identified a total of 50 (Supplementary Table S6) polar metabolites, which correspond to soluble sugars (9), soluble alcohols (5), organic acids (8), amino acids (20) and other compounds (7) (Figure 6; Supplementary Tables S5 and S6). Significant alterations in the metabolites between *Pvgstu3–3.4* and WT plants under control and combined stress conditions were observed due to genotypic (66%), treatment (80%) and treatment × genotype interaction (60%) effects, respectively.

Overexpression of the *Pvgstu3–3.4* had a significant effect on the metabolic profile of transgenic plants under nonstressed conditions as indicated by the 23 out of 50 metabolites being significantly different from the WT plants, 20 of which were downregulated and only three were upregulated (Table 4; Figure 6). The metabolites that were downregulated were mainly soluble sugars (five), such as sucrose, fructose and glucose, amino acids (nine), such as valine, proline, oxoproline, the soluble alcohols mannitol, sorbitol and myo-inositol and the organic acids malic and glyceric acid.

The effect of severe combined DT stress on the WT plants induced significant alterations in 30 out of the 50 metabolites, of which 28 were upregulated, and sucrose and myo-inositol were downregulated compared to the stress-free plants (Table S6). Soluble sugars, such as galactose and fructose were greatly increased (approximately 700- and 10-fold respectively), along with organic acids, such as 2-oxoglutaric acid and the TCA cycle intermediate citric acid (160- and 46-fold respectively) and amino acids, such as valine, isoleucine, proline (249-fold) and oxoproline (116-fold), lysine (523-fold) and tryptophan (93.7-fold).

**Figure 7:**
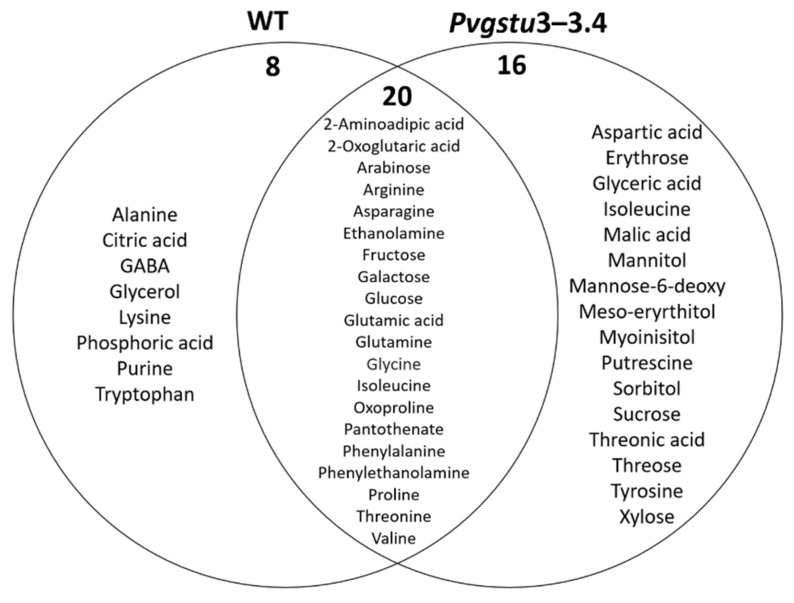
Venn diagram representation of metabolites commonly or differentially increased in the leaves of WT and *Pvgstu3–3.4* tobacco plants grown for 9 days under combined drought and heat stress (DT) compared to stress-free plants.

The metabolic response of the *Pvgstu3–3.4* plants revealed that more metabolites (37) were significantly altered in comparison to WT plants under combined drought and heat (DT) stress treatment (Figure 7; Table S6). *Pvgstu3–3.4* plants upregulated 36 metabolites, of which 9 were soluble sugars (mainly mannose-6-deoxy, sucrose, galactose, glucose, fructose), four soluble alcohols (mainly mannitol, sorbitol), five organic acids (such as glyceric, 2-oxoglutaric and malic acids) and 14 amino acids (mainly proline and oxoproline) and downregulated only nicotine. Further comparison showed that 16 metabolites are unique to *Pvgstu3–3.4* under stress combination, including the compatible solute mannitol involved in osmoregulation and sugars, such as sucrose mannose-6-deoxy and erythrose (Figure 7; Table S6).

The 14 metabolites that contributed to the distinction between *Pvgstu3–3.4* and wild type plants under combined DT stress conditions were mainly upregulated, 4 of which were soluble sugars (fructose, glucose, sucrose and xylose), 2 were soluble alcohols, including sorbitol (2.1-fold) and myo-inositol (2.9-fold), threonic organic acid and amino acids, including arginine (20.5-fold), glycine (3.2-fold), tyrosine (2.2-fold) and proline (1.3-fold) (Table 5).

## 3. Discussion

The effect of combined abiotic stresses on plant responses remains elusive, despite the importance of developing crops able to acclimate and be productive in a rapidly changing environment [6]. Studies on combinations of drought and heat stresses have revealed that physiological and molecular responses of plants subjected to combined stress are different from that of the single stresses [5,10]. GSTs are key antioxidant enzymes that can be induced by diverse environmental stimuli [15]. Overexpressing *gst* genes in plants has elucidated their role in response mechanisms contributing to abiotic stress tolerance [15]. Up to date, the role of *gst* genes from the common bean and, in particular, the EU PGI variety of “Plake Megalosperma Prespon” has not yet been investigated. In this work, we have used two genes, isolated from leaves (*Pvgstu2–2*) and roots (*Pvgstu3–3*) of *P. vulgaris* L., for their high antioxidant catalytic function [21,33]. Our results show that overexpression of both *Pvgstu* genes in tobacco enhances plant tolerance to single heat stress and the *Pvgstu3–3.4* to a combination of heat and drought stress. The metabolic and whole-genome transcript profiling identified stress-induced pathways that possibly contribute to plants’ performance under stress conditions.

A single or combined abiotic stress response in plants involves changes in morphological, physiological and biochemical properties [2]. Underwater limitation, the leaf fresh and dry weights are significantly reduced [37], and low photosynthetic rate and reduced turgor pressure restrict leaf extension [38]. Herein, the combined stress treatment was more severe compared to single stress effects, and *Pvgstu3–3.4* was more tolerant compared to *Pvgstu2–2.19* and WT plants. We observed a similar morpho-physiological response pattern between *Pvgstu3–3.4* and WT plants in nine days under single drought and combined DT stress, manifested as a reduction in M_F_ under single D and DT stresses and loss of leaves under severe DT stress. However, the differentiated response was the maintenance of root length in *Pvgstu3–3.4* even under combined DT stress compared to a severe approximately 54% reduction observed in WT plants. Growth response after 16 days in single heat stress (T) was also greater in *Pvgstu3–3.4*, showing increased root length and M_F_, but we did not observe any significant differences between genotypes under single drought stress (D). This may indicate that either the duration of drought did not trigger any responses or that oriental tobacco cv Basmas Xanthi may have acquired acclimation over time as it is a variety cultivated in drought-prone areas. These results support the observations from previous studies, which have demonstrated an increase in the growth of plants overexpressing GSTs under salt stress [27,39,40]. It is worth mentioning that when severe DT treatment induced significant wilting on day 9 (mid-harvest), plants were re-watered but remained under heat stress to assess whether the wilting was associated with drought or heat stress, given that plants under single D or T stresses did not show any significant wilting. All plants effectively recovered compared to the DT plants on day 16 for all transgenic and WT plants indicating that despite the non-significant effect of single drought treatment on the morphological parameters, it showed a predominant negative effect over heat stress when the stresses were combined (DT). Similar results were observed in tomatoes under individual heat and drought and their combination [41]. *Pvgstu3–3.4* increased photosynthetic efficiency as an early response to single heat (T) and drought (D) stresses after 16 days compared to WT plants. Interestingly, the *Pvgstu2–2.19* transgenic line also showed a greater maximum quantum yield of PSII under single drought stress (D) compared to WT. Studies have shown that reduction in photosynthetic activity is a common response to single heat and drought stresses; nevertheless, photosynthesis is less affected by heat stress, and only temperatures over 40 °C are known to have a detrimental effect [10]. Similarly, heat stress did not reduce the photosynthetic activity of tobacco plants, but drought stress and combined heat and drought stress led to more than 80% reduction in photosynthetic activity [4]. Herein, combined DT stress led to a significant reduction in photosynthetic activity on day 9, and no measurements were able to be performed on day 16 due to extensive wilting for both transgenic lines and WT plants.

The morphophysiological results indicate the contribution of *Pvgstu3–3.4* overexpression to possible thermotolerance, expressed as greater plant performance under heat stress and higher leaf number under combined DT stress compared to WT. Overexpression of HSPs in tobacco plants has led to increased tolerance to heat stress [42]. Thus, far, there are limited reports on GSTs contribution to thermo-tolerance, despite the evidence for cold-induced GSTs demonstrated in *Brassica oleracea* [43], *Cucurbita maxima* [44], overexpression in *Arabidopsis* [45] and in rice [46], whereas in transgenic tobacco [47] resulted in enhanced tolerance to high temperature. The combined treatment effect was too severe for the photosynthetic capacity of both transgenic lines and WT plants after 16 days of combined drought and heat (DT) stress. Similar results were observed in transgenic tobacco plants subjected to short-term (5 days) drought and one additional day of heat stress, where it was shown that single heat stress alone did not affect the plants significantly but intensified the effect of drought when combined [24].

We then proceeded with analyzing the molecular alteration that occurred in the *Pvgstu3–3.4* transgenic plants under control and combined DT conditions in comparison to WT under control conditions aiming at investigating the molecular mechanism underlying the stress-tolerance phenotype of the transgenic plants. Thus, we analyzed, through next-generation sequencing, the entire transcriptome of the *Pvgstu3–3.4* and WT plants under control and combined DT conditions. The comparison between the WT plants and *Pvgstu3–3.4* under stress-free conditions revealed significant changes in the transcription profile of *Pvgstu3–3.4* even before the application of the stress. More specifically, the comparison of WT and transgenic tobacco plants overexpressing *Pvgstu3–3.4*, prior to the application of concurrent DT stress, indicated that in the metabolism of xenobiotics by cytochrome P450 pathway, the gene encoding cytochrome P450 family 1 (which is an uncharacterized monooxygenase) is overexpressed in the GST plants, and this gene is also involved in tryptophan metabolism. This is in accordance with [48], where the overexpression of a P450 (CYP) 714 protein family monooxygenase in rice provided enhanced tolerance to salt stress. The NF-kappa B signaling pathway has been shown to be implicated in drought response [49]; herein, the 2.8-fold upregulated ubiquitin-conjugating enzyme E2 I (UBC9) genes in the *Pvgstu3–3.4* have been previously reported to play an important role in plant response to heat [50] and drought [51,52] stresses. Furthermore, gene expression alterations were detected in the linoleic acid metabolic pathway and, more specifically, the gene encoding linoleate 9S-lipoxygenase that can use linoleic acid or linolenic acid as substrates. Plant lipoxygenases are considered to play a role in plant physiology like growth and development, as well as in pest resistance and senescence or response to wounding. In *Arabidopsis thaliana* seedlings, treatment with 9-Hydroxyoctadecatrienoic acid (the byproduct of lipoxygenase action) resulted in a reduction in lateral roots and activated events common to developmental and defense responses. Yet, the downregulation of the gene may suggest that the overexpression of the *Pvgstu3–3.4* compensates the action of this lipoxygenase, at least with regards to stress-tolerance [53]. The acylglycerol lipase gene was also found to be upregulated in the glycerolipid metabolism, suggesting an increase in glycerol, which is the backbone of triacylglycerols. In *A. thaliana,* under heat stress, an increase in triacylglycerol levels was observed, which operates as an intermediate of lipid turnover, and results in a decrease in membrane polyunsaturated fatty acids [54]. The gene for phospholipase D1/2 in the phospholipase D signaling pathway was downregulated, which may lead to reduced cell proliferation and differentiation. This finding is in accordance with Distéfano et al. [55], showing that phospholipase D1/2 mutants may behave as drought-primed, leading to their tolerance under severe drought stress. The above results suggest that the *Pvgstu3–3.4* transgenic plants may be in a stress-tolerant primed condition.

Regarding the changes in transcriptomes between stress-free WT plants and transgenic *Pvgstu3–3.4* under DT at 48 h, we observed that glutathione S-transferase and glucuronosyltransferase genes were upregulated in the metabolism of xenobiotics by the cytochrome P450 pathway in the transgenic plants. This is not surprising as the combined DT stress is severe and possibly triggers a plant response through activating the detoxification systems like GST and P450, which play a prominent role in the crosstalk between abiotic stress responses [56]. The involvement of GSTs in stress-tolerance is well documented [18,27,57,58,59,60,61,62,63,64]. Comparison between the stress-free and combined DT stress conditions after 48 h in the transgenic line *Pvgstu3–3.4* showed that similar to the stress-free condition, genes encoding enzymes in the metabolism of xenobiotics by cytochrome P450, such as GST and glucuronosyltransferase, as well as SOD and Catalase, were found to be upregulated in *Pvgstu3–3.4* plants after 48 h of stress-induction. SOD and CAT enzymes are well-known enzymes involved in the detoxification of organisms from ROS [65,66,67]. This may be due to the severity of the combined stress, suggesting that GST overexpression on its own may not be capable of detoxifying the plant and thus, an increase in other detoxification enzymes is required. Under DT stress, the *Pvgstu3–3.4* showed enhanced carbon metabolism, despite being considered to be affected by water limitation and increased temperatures [68]. Adaptation in primary metabolic pathways, such as the TCA cycle, may contribute to the protective role against oxidative stress through maintaining C/N balance by converting glutamate in the cytosol to succinate and by generating NADH and succinate [69], as it was observed in the *Pvgstu3–3.4* plants under DT conditions.

An increase in the expression of genes in the photorespiration pathway was observed under DT conditions, which is considered to protect photosynthesis from photoinhibition and prevent ROS accumulation in leaves [70], indicating preservation of the photosynthetic efficiency under the concurrent occurrence of drought and heat stress. In the fructose and mannose metabolism, as well as glycolysis/gluconeogenesis pathways, the 6-phosphofructokinase 1 (PFK1) coordinating the photosynthetic carbon flow into sucrose and starch biosynthesis [71] was upregulated in *Pvgstu3–3.4* under DT at 48 h. Another enzyme involved not only in glycolysis/gluconeogenesis but also in pyruvate metabolism responsible for increased pyruvate is pyruvate kinase, which was increased in *Pvgstu3–3.4* line under combined DT treatment. Pyruvate kinase has been found to be increased during mild stress [72] and during heat stress in fungus *Metarhizium robertsii* as the first line of defense [73]. The conversion of pyruvate to acetyl-CoA in the chloroplasts is the start of the fatty acid biosynthesis, such as Triacylglycerol (TAG), which are linked to plant stress response (Lu et al. 2020). Herein, the genes involved in the generation of acetyl-CoA, the key intermediate in a number of different metabolic pathways (Oliver et al. 2009), were upregulated under DT conditions in the *Pvgstu3–3.4* plants.

The ATP-binding cassette (ABC) transporters, which among others are involved in the detoxification process, transport of the phytohormones auxin and abscisic acid and maintaining homeostasis under abiotic stresses (Dajuja et al. 2020), herein were upregulated under DT conditions (48 h) in *Pvgstu3–3.4*. Similarly, the indole-3-glycerol phosphate (IGP) of the tryptophan (Trp) biosynthetic pathway was upregulated, which may play a central role in the Trp-independent IAA de novo biosynthetic pathway as it was previously shown in *A. thaliana* [74] and therefore, in signal transduction pathway under stress conditions [75]. Soluble sugars were highly sensitive to environmental stresses and, most importantly, sucrose and hexoses, which are involved not only in the regulation of growth-related genes but also in downregulation of stress-related genes as reviewed by Rosa et al. [76], suggesting again that the plant may be in a primed condition due to the overexpression of a *Pvgstu3–3.4*. The same applies to the maltase-glucoamylase, which was upregulated under DT conditions in *Pvgstu3–3.4* transgenic plants, suggesting increased biosynthesis of fructose and beta-glucosidase and maltase-glucoamylase leading to glucose biosynthesis; in addition, alpha-amylase was also upregulated, potentially leading to enhanced maltose biosynthesis [76].

Transcription factors (TFs) are key regulators of signal transduction and gene expression by modulating essential aspects of plant function, including plant adaptation to adverse environments [77,78,79]. The early expression of MYB, MYB-related, AP2-EREBP, NAC, HSF, and WRKY TF families in *Pvgstu3–3.4* plants even in stress-free conditions and their increase under DT stress-indicate that the *Pvgstu3–3.4* transgenic plants were in a primed condition prior to the induction DT stress. The MYB TFs are known to be involved in the transcriptional control of physiological and biochemical processes, including plant development, cell fate determination, and secondary metabolism along with mediating abiotic stress responses [78,80], such as during heat stress in eggplant [81] or combined heat and drought stress in soybean [82]. Ethylene-responsive element-binding protein (AP2-EREBP) TF family is involved in the biotic and abiotic stress response via the Jasmonic acid pathway and ABA biosynthetic pathway, respectively [83] and have been shown to be involved in early-stage drought stress responses [84,85,86]. NAC TFs are also involved in both abiotic and biotic stress responses [78], and herein they were both presents in stress-free conditions and to a greater extent under DT stress. Heat shock transcription factors (HSF) exert a central regulatory role in alleviating the negative effects of not only high temperature [87,88] but also drought and salt stress [87]. The WRKY family is a key component of pathways, such as carbohydrate synthesis, senescence, development, and secondary metabolites synthesis and also of the plant–pathogen interaction pathway [89]. Herein, despite being mainly downregulated under increased exposure to DT stress, confirming the negative effect of temperature stress on WRKY genes expression as an energy maintenance mechanism [88], they were also present under stress-free conditions in *Pvgstu3–3.4* plants, which could be related to the biotic induced nature of the *Pvgstu3–3.4* gene. Other stress-related TFs were among the top 15 TF families expressed under DT stress in *Pvgstu3–3.4*, such as the FAR- RED-IMPAIRED RESPONSE1 (FAR1), MADS, Trihelix, ARF, ARR-B, SBP, bZIP and Sigma70-like. FAR1 directly facilitates the light-induced inositol biosynthesis, which would otherwise be produced indirectly via photosynthesis, and suppress light-induced oxidative stress [90], indicating that FAR1 TFs may have played a protective role in the photosynthetic capacity of the *Pvgstu3–3.4* plants under DT stress. Another TF family involved in light response mechanisms is the Trihelix family, which also involves responses to salt and pathogen stresses [91], cold and salt stresses [92] and salt, freezing and drought stress [93]. Auxin response factors (AFR) have been characterized as regulators of the auxin role in tomato response to high salinity and water deficit [94], which may be associated with the enhanced transcription of indole-3-glycerol phosphate (IGP) in the tryptophan (Trp) biosynthetic pathway and the Trp-independent IAA de novo biosynthetic pathway observed in the *Pvgstu3–3.4* plants. Basic leucine zipper (bZIP) TFs have significant roles in the regulation of ABA-related signaling pathways [77] and are involved in abiotic stresses, such as drought, salinity, and heat stress [95]. Interestingly, a root-specific bZIP identified in both the drought-resistant tepary bean (*Phaseolus acutifolius*) and drought-sensitive common bean was shown to be responsive to water deficit stress possibly by coordinating gene expression [96], considering that root phenology/physiology is an essential component in *P. vulgaris* for performance under drought stress [97,98,99]. The significant upregulation of the Type B ARR TF family only under DT conditions in *Pvgstu3–3.4* plants indicates the involvement of cytokinin signaling in response to DT stress, which in conjunction with other phytohormones at different regulatory layers [100], may be involved in the signal transduction under the combined DT stress. Similarly, Sigma70- like TFs were also upregulated under DT conditions in *Pvgstu3–3.4*, which confirms their role as potential regulators of plant responses to various abiotic stress conditions [101].

By increasing the accumulation of compatible solutes, metabolic acclimation is considered a key mechanism for the protection and survival of plants under abiotic stresses. The metabolic analysis showed that overexpression of *Pvgstu3–3.4* might have a signaling role that was mainly stimulated under stress conditions in order to increase specific metabolites and activate the protective mechanisms, while metabolites, such as proline, oxoproline, mannitol, and sorbitol, malic acid and soluble sugars were downregulated in stress-free conditions. The accumulation of a wide range of metabolites, such as soluble sugars and amino acids, can enhance abiotic stress-tolerance and restore homeostasis [102,103]. *Pvgstu3–3.4* line under combined heat and drought stress has demonstrated a distinct protective effect to the plant homeostatic mechanism by upregulating metabolites, such as the compatible solute mannitol involved in osmoregulation and sugars like sucrose and mannose-6-deoxy, a cellular redox regulator and precursor of ascorbic acid, sorbitol, malic acid and aspartic acid. The osmoprotectant mannitol was differentially increased in the *Pvgstu3–3.4* plants in response to combined DT stress, but not in the WT plants. Mannitol, like other polyols, has been shown to protect membranes and enzyme complexes from ROS mainly through the glutathione–ascorbate cycle or cell signaling pathways during stress [104]. The expression of mannitol-1-phosphatedehydrogenase (*mtlD*) and the accumulation of mannitol in the chloroplast enhanced tolerance of transplastomic tobacco plants to oxidative stress [105]. However, transgenic tobacco plants overexpressing a *Gm*GSTU4 decreased mannitol concentration under salinity stress. The observed increase in myo-inositol in *Pvgstu3–3.4* compared to WT plants under combined stress treatment also has been shown to be accumulated under most of the abiotic stresses and is considered to contribute as a compatible solute to stress-tolerance [106].

Sorbitol is a photosynthetic compound produced in mature leaves and, along with sucrose, both translocate carbon and energy between sources and sink organs. Sorbitol content has been shown to increase in response to drought stress in *Arabidopsis* [107] and tobacco [108]. Accumulation of the osmoprotectant proline is also positively correlated with drought and salinity tolerance [18]. Increased accumulation of proline and sorbitol was observed by Kissoudis et al. [109] in tobacco plants overexpressing a *Gm*GSTU4 under salinity stress. Overexpression of a GST gene from the halophyte *Limonium bicolor* also enhanced proline accumulation in tobacco plants under salt stress [39]. In contrast to our results, drought-induced proline did not accumulate under combined drought and heat stress in *Arabidopsis* and soluble sugars, such as sucrose, maltose and gulose, were increased instead [5], suggesting that during the combination of drought and heat stress, sugars replace proline in plants and functions as major osmoprotectants [110] to avoid proline toxicity in the mitochondria [5]. However, the involvement of proline in the protection of plants against combined drought and heat stress has been recently demonstrated [111]. The results of this study indicate a distinct protective role of *Pvgstu3–3.4* to the plants’ homeostatic mechanisms under combined drought and heat stress. The significant, but moderate increase in proline (1.3-fold) and the greater accumulation of sugars (5.3- and 4-fold sucrose and glucose, respectively) identified herein, may be, in fact, maintaining the turgor pressure against osmotic stress and mitigating the negative effects of oxidative stress contributing to the stabilization of the photosystem II complex [112] and restore energy homeostasis [113].

Plant acclimation and maintenance of yield in response to combined abiotic stresses require the activation of multiple signaling pathways to regulate stress-inducible genes involved in stress-tolerance [114]. Previously, novel biotic-stress-inducible *Pvgstu3–3.4* has been isolated from roots and herbicide induced *Pvgstu2–2* from the leaves of *P. vulgaris*. Herein, the morphophysiological results showed potential thermotolerance of the transgenic lines overexpressing the *Pvgstu* genes, with the *Pvgstu3–3.4* also showing enhanced tolerance to the combination of drought and heat stress. This effect was further investigated by the transcriptomic and metabolic profile of *Pvgstu3–3.4* plants subjected to combined drought and heat stresses, exhibiting a unique acclimation response. The greater number of differentially expressed genes, specifically altered during combined DT stress, is in accordance with the results of [6] that the transcripts tend to be greater in number under combined abiotic stresses. Enzymes, such as GSTs, CAT and SOD, were overexpressed in the transgenic plants along with other enzymes involved in plant tolerance mechanisms. The enhanced early protective response of *Pvgstu3–3.4* to combined heat and drought stress as a result of the upregulation of central metabolism, as levels of sugars, amino acids and TCA cycle intermediates were generally increased. Regulation of certain genes and the accumulation of specific metabolites indicates that the transgenic plants were probably in a stress-primed condition. The overexpression of *Pvgstu3–3* may also play a signaling role in the plant that is mainly stimulated under severe stress in order to increase specific metabolites and activate the protective mechanisms. Aside from the protective role of *Pvgstu3–3* attributed to the observed increased metabolism levels, the interaction of *Pvgstu3–3* with the secondary metabolites could be partially attributed to the ligandin functions that several GST show [115,116]. However, further research is required to determine if any ligand-binding interactions exist for this particular GST isoenzyme.

Recently, Zandalinas et al. [11] demonstrated that plants subjected to stress combination could integrate different systemic signals and the most efficient manner in which plants perceive the different stresses is when the signals of the two stresses originate from distant parts of the plant. Similarly, under heat and drought combination, the signals originate from different parts of the plant, i.e., above- and below-ground, respectively, and based on the results presented herein, different systemic signaling pathways were activated, possibly inducing an acclimation process [117]. Major components of the regulatory networks are shared between abiotic and biotic stress signaling [118,119], including the production of ROS [120,121], calcium fluxes [122], transcription factors [78], growth regulators [123] and mitogen-activated protein kinase (MAPK) cascades [123]. Moreover, redox state, as well as metabolic compounds, have a major role in post-translational modifications, regulating the activity of target proteins and transcription factors [119]. Transcription factors, such as WRKY, MYB, ERF, NAC, bZIP and HSF that were also identified in the transgenic *Pvgstu3–3* under DT stress are involved in the regulation of stress crosstalk and are considered to have similar induction patterns across various abiotic and biotic stresses [78]. In this study, we showed how a biotic-inducible *Pvgstu3–3* could activate early protective response mechanisms under a combination of drought and heat stress. Such response involved complex crosstalk among different regulatory levels, including adjustment of metabolism and gene expression for biochemical and molecular adaptation.

## 4. Materials and Methods

### 4.1. Plasmid Constructs and Agrobacterium-Mediated Transformation

The *Pvgstu2–2* and *Pvgstu3–3* genes were isolated from two different parts of the *P. vulgaris* var. Plake Megalosperma Prespon (Florinas) of protected geographic indication (PGI) due to their induced expression in the different organs, i.e., in the leaves (*Pvgstu2–2*) after herbicide application and the roots (*Pvgstu3–3*) after exposure to biotic factor [17,26], respectively. Standard recombinant methods were implemented for the development of plasmid constructs after ligation into a pART27 expression vector. Ligase reactions were performed according to the New England Biolabs protocol. Primers used in PCR were for the *Pvgstu2–2*, the forward 5′-GAGCTACTGCAAAGCCCTTTGTTTG-3′ and the reverse 5-′CATTAGAATGAACCGAAACCGGCGG-3′, while for the *Pvgstu3–3* the 5′-GAGAGCTCGAAAGTGAGGGTATTGGG-3′ and 5′-CATTAGAATGAACCGAAACCGGCGG-3′.

The resulting expression constructs (pART27-*Pvgstu2–2* and pART27-*Pvgstu3–3*) were used to transform competent *E. coli* cells (strain Μach 1). The plasmid DNA of the verified positive clones was isolated with the PureLink ™ Quick plasmid miniprep kit (Invitrogen, Carlsbad, CA, USA). Plasmid pART27-*Pvgstu2–2* was digested with EcoRI and BamHI, while pART27-*Pvgstu3–3* was digested with HindIII. The same restriction enzyme reactions were performed on the pART27-*gstu*4 vector [124] used as a negative control. Reactions were performed in a 10 μL final volume mixture containing 1X restriction enzyme buffer, 180 ng μL plasmid DNA, 20 U μL EcoRI HF and BamHI or HindIII HF (New England Biolabs, Ipswich, MA, USA) and were incubated at 37 °C for 60 min. The products of the digestion reactions were electrophoresed on 1% (*w/v*) agarose gels in TBE solution. The selected clones were sequenced, and the analysis of the results was performed with the SeqMan Pro program. Positive colonies were amplified in liquid cultures followed by isolation of the plasmid DNA with the PureLink ™ Quick Plasmid Miniprep (Invitrogen, Carlsbad, CA, USA) kit and were cloned into *Agrobacterium tumefaciens* cells (strain GV3101) using the electroporation method.

*Agrobacterium*-mediated transformation was used to develop transgenic tobacco (cv. Basmas Xanthi) lines overexpressing the *Pvgstu3–3* and *Pvgstu2–2* genes. The co-cultivation phase involved the growth of *A. tumefaciens* in 5 mL liquid LB substrate supplemented with kanamycin (20 mg L^−1^), spectinomycin (100 mg L^−1^) and gentamicin (50 mg L^−1^) at 28 °C in a shaking incubator (1035 g) for 3 days, in order to select for bacteria carrying the modified plasmids pART27-*Pvgstu2–2* and pART27-*Pvgstu3–3*. Following centrifugation at 11,200 g for 5 min, the bacterial cells and tobacco leaf discs (~1 cm^2^ diameter) were placed in MS supplemented with 0.1 mg L^−1^ ΝΑΑ and 1 mg L^−1^ BAP (MSR1) at 28 °C in a shaking incubator (1035 g) for 30 min. For the regeneration, the leaf discs were transferred in the selection media (MSR1 supplemented with 50 mg L^−1^ kanamycin + 250 mg L^−1^ cefotaxime).

### 4.2. Verification of Putative Transgenic Lines, Relative Expression of the 35S-Pvgstu2–2 and -Pvgstu3–3 and Enzymatic Activity

Genomic DNA from the putative transgenic T0 lines of *Pvgstu3–3* (10 independent lines) and *Pvgstu2–2* (21 independent lines) was isolated using the NucleoSpin^®^ Plant II (Macherey–Nagel). The real-time PCR (RT–PCR) reactions were carried out with the following forward 35S 5′-GCTCCTACAAATGCCATCA-3′ and reverse nos-ter 5′-TTATCCTAGTTTGCGCGCTA-3′ primers in a Rotor-Gene 6000 real-time 5-Plex HRM PCR thermocycler (Corbett Research, Sydney, Australia), using the Rotor-Gene Q software version 2.0.2 (Corbett Life Science, Cambridge, UK). PCR reaction mixtures consisted of 50 ng genomic DNA, 1X PCR buffer, 1.5 mM MgCl_2_, 0.5 μM of the forward and reverse primers, 0.2 mM deoxyribonucleotide triphosphate (dNTP) mix, 0.5 mM SYTO^TM^ 9 green fluorescent nucleic acid stain (Invitrogen, CA, USA), and 1 U Kapa Taq DNA polymerase (Kapa Biosystems, Cape Town, South Africa). The amplification was performed as follows: initial denaturation at 95 °C for 2 min, followed by 30 cycles of 95 °C for 20 s, 54 °C for 20 s, and 72 °C for 1 min with a last melting step at 65–95 °C.

The selection of the positive for the transgene T0 lines was based on: i) the relative expression level of the transgene obtained through quantitative reverse transcription PCR (q-RTPCR) analysis and ii) the activity of *Pv*GSTU*2–2* and *Pv*GSTU*3–3* enzymes. Total RNA was extracted using TRIZOL (Invitrogen, Carlsbad, CA, USA). First-strand cDNA was synthesized (0.1 μg) using the SuperScript II reverse transcriptase kit (Invitrogen, CA, USA) and 10 U Superscript II reverse transcriptase according to the manufacturer’s instructions. Primers used for q-RTPCR analysis were the forward 5′-GGGCAACAAGAGTGAACAGC-3′ and the reverse 5′-TCCACGTAGCACCCACAATC-3′ for the *Pvgstu2–2*, and the forward 5′-GGAAGTGTAAAGAAAAATGGCAGAGCA-3′ and reverse 5′-GACTCAGCAATGGCCTTTCC-3′ for the *Pvgstu3–3* transgenes. Primers used for *β-actin* reference gene were the 5′- GGTGACGAACTCAGTCCAAAAGGGGT–3′ as forward and 5′-ACGGCCACTGGCGTATAGGGACAACA -3′ as the reverse. PCR reaction mixtures consisted of 50 ng cDNA, 1X PCR buffer, 1.5 mM MgCl_2_, 0.5 μM of the forward and reverse primers, 0.2 mM dNTP mix, 0.5 mM SYTO^TM^ 9 green fluorescent nucleic acid stain (Invitrogen, Carlsbad, CA, USA), and 1 U Taq polymerase (Kapa Biosystems, Cape Town, South Africa). The amplification was performed in a Rotor-Gene 6000 real-time 5-Plex HRM PCR Thermocycler (Corbett Research, Sydney, Australia) with 3 technical replications per sample as follows: initial denaturation at 95 °C for 2 min, followed by 30 cycles of 95 °C for 20 s, 54 °C for 20 s, and 72 °C for 1 min with a last melting step at 65–95 °C. Five μL of reaction products were separated on 2% agarose gel, stained with ethidium bromide and visualized on UV. The expected fragment for the *Pvgstu2–2* gene was 148 bp, whereas for the *Pvgstu3–3* gene was 220 bp and 345 bp for the β-actin gene. The results were presented as a relative quantitative expression based on the 2^-ΔΔCT^ method [125].

The enzymatic activity of the 9 T0 lines overexpressing the *Pvgstu3–3* was assessed towards the 1-chloro-2,4-dinitrobenzene (CDNB) universal nonspecific GST substrate that showed the highest sensitivity and specificity (3.5 U mg^−1^), as described in Chronopoulou et al. [21]. However, some GSTs show limited activity on this substrate and, as an alternative, the 7-chloro-4-nitrobenz-2-oxa-1,3-diazole (NBD-Cl), given its involvement in the GSH complexation reactions. Therefore, the enzymatic activity of the 8 T0 lines overexpressing the *Pvgstu2–2* was assessed towards only the NBD-CI, in which this isoenzyme exhibits high activity of 148.2 U mg^−1^ [33] according to [126,127]. The measurement of the specific enzymatic activity of the isoenzymes *Pv*GSTU*2–2* and *Pv*GSTU*3–3* was performed in leaf extract from T0 genetically transformed tobacco lines and WT plants used as negative controls. The first two fully expanded leaves were homogenized in a 5 mL aqueous solution of 100 mM Tris-HCl (pH 7.5) and 5% (*w/v*) polyvinylpyrrolidone (PVP) using a blender. After centrifugation at 15,000×g for 15 min at 4 ^°^C, the supernatant was used for the enzymatic assays. GST activity towards CDNB was determined at 340 nm and towards NBD-Cl at 420 nm. The enzymatic activity was assessed spectrophotometrically and expressed as specific activity (SA Units mg^−1^%).

A sufficient number of independent transgenic lines were hardened and transferred in greenhouse conditions to produce the T1 generation. To avoid cross-pollination, the inflorescences were covered with paper bags shortly before the flowers bloomed. The harvest was performed by cutting the inflorescences after seed maturation and drying of the capsules was completed. The T1 generation was then used to study the involvement of *Pvgstu2–2* and *Pvgstu3–3* in abiotic stress tolerance, and more specifically in a combined and single heat and drought stress conditions.

### 4.3. Application of Abiotic Stress Treatments and Morphophysiological Measurements

The seeds of the T1 lines were initially grown in a selection MS medium supplemented with kanamycin (100 mg L^−1^), and the WT tobacco seeds were placed in a plain MS medium. After selection, the plantlets were transferred in MS for further growth, and when they reached four true leaves, the rooted plants were transplanted into pots containing 2:1 peat:perlite and transferred for hardening in a CRW- 500 SD growth chamber (Chirisagis, Athens, Greece) with approximately 23 ± 0.1 ^°^C After two weeks, plants of the two different lines were subjected to stress-free (23 ^°^C, well-watered-control; C), drought (23 ^°^C and water deprivation; D), high temperature (38 ^°^C, well-watered; T) and a combination of high temperature and drought (water deprivation and 38 ^°^C; DT) conditions in a controlled growth chamber environment. The watering for the plants under C and T treatments was performed every 2 days until saturation. The photoperiod was set at 12 h, from supplemental lighting of cool-white lamps with an average of 400 μmol photons m^2^ s^−1^ photosynthetically active radiation.

The experimental design was completely randomized, with *n* = 8 for the WT plants and *n* = 18 for the two transgenic lines for each treatment. The duration of the experimental period was 16 days. The physiological measurements were performed at 0, 8 and 24 h, and on days nine (mid-harvest) and 16 (final harvest). The transgenic (*n* = 5) and WT (*n* = 4) plants that were exposed to the combined stress treatment (DT) were re-watered on day 9, due to extensive wilting, to assess their recovering efficiency, and the plants remained under heat to test whether the wilting was related to drought or heat stress. The morphological measurements of all treatments were assessed at mid- and final harvests, whereas recovery was assessed only for the DT treatment on the final harvest day (day 16). Regarding the physiological measurements, plants under combined DT treatment were not measurable on the final harvest day due to severe wilting, and, therefore, all measurements for DT were performed at 9 days and were compared with the recovery state at 16 days. Leaf samples for transcriptomics and metabolomics analysis were excised at 0, 8 and 48 h, and on day 9, respectively. Relative chlorophyll content was measured on one leaf in three technical replications averaged for each plant as described in [125] using a CCM-200 plus chlorophyll content meter (Opti-Sciences Inc., Hudson, NY, USA). Dark-adapted chlorophyll *a* fluorescence analysis was performed on the adaxial leaf surface using the OS30p+ chlorophyll fluorometer (Opti-Sciences Inc., Hudson, NY, USA) after 30 min dark adaptation. Harvested plants were separated into leaves, stems and roots, and the final morphological parameters, such as the stem and root length, number of leaves and fresh plant matter (M_F_), were measured. The plant dry matter (M_D_) was obtained after drying at 60 °C until constant weight.

### 4.4. Total RNA Extraction, Library Preparation and Sequencing

Total RNA was extracted from leaf samples of the best performing line (*Pvgstu3–3.4*) (*n* = 6) under combined DT stress at 0, 8, and 48 h and from the WT (*n* = 2) at 0 h using the Monarch Total RNA Miniprep kit (New England BioLabs Ltd., Ipswich, MA, USA), according to manufacturer’s instructions. The integrity of the RNA was evaluated using a 2100 Agilent Bioanalyzer and the Agilent RNA 6000 Nano Kit (Agilent Technologies, Santa Clara, CA, USA), and the RNA concentration was determined using the NanoDrop^TM^ (Thermo Scientific, Waltham, MA, USA). Following purification, the mRNA was fragmented and copied into the first-strand cDNA using reverse transcriptase and random primers. After the second strand cDNA synthesis and the addition of a single “A” base and subsequent ligation of the adapter, the products were purified and enriched with PCR amplification. The PCR yield was quantified with Qubit and was subjected to single-strand circularized DNA molecule (ssDNA circle) preparation for the final library. Each cDNA library was sequenced in a single lane of the BGISEQ-500 system with a paired-end sequencing length of 100 bp according to the manufacturer’s instructions at the Beijing Genomics Institute (BGI, Shenzhen, China). The transcriptome data in Table 6 were compared as follows: WT plants under control conditions (group 1) versus *Pvgstu3–3* under control (group 2), DT at 8 h (group 3) and DT stress at 48 h (group 4). A comparison between the *Pvgstu3–3* under stress-free (group 2) and combined stress at 48 h (group 4) was also performed.

#### Transcriptomics Data Processing and Functional Enrichment Analysis

The raw reads were filtered using SOAPnuke software. The clean reads were mapped onto the reference genome, followed by novel gene prediction, SNP and INDEL calling and gene-splicing detection. More specifically, the hierarchical indexing for spliced alignment of transcripts (HISAT) pipeline was used to align the reads against the reference genome [128]. The StringTie [129] was applied for transcript reconstruction and Cuffcompare (Cufflinks tools) [130] to compare reconstructed transcripts to reference annotation. The coding potential of novel transcripts was predicted by CPC [131] and was merged with reference transcripts to get a complete reference on which the downstream analysis was based. The clean reads were mapped to reference using Bowtie2 [132], and then the gene expression level was calculated with the software RNA-Seq by Expectation Maximization (RSEM) [133]. The detection of DEGs was performed with NOIseq, as described in Tarazona et al. [134]. The hierarchical clustering analysis of DEGs was performed using the *pheatmap* function in R. The subsequent GO annotation was performed and GO functional enrichment was applied using p hyper in R. The pathway analysis involved the Kyoto Encyclopedia of Genes and Genomes (KEGG) annotation analysis and the pathway functional enrichment using *phyper* in R. For the transcription factor prediction of DEGs, the *getorf* function was used to find the open reading frame (ORF) of each DEG, which was aligned to TF domains using *hmmsearc*h [135]. For the protein–protein interactions (PPI) analysis of DEGs, the DIAMOND [136] was used to map the DEGs to the STRING database [137] to obtain the interaction between DEG-encoded proteins using homology with known proteins.

### 4.5. Metabolite Extraction and GC–MS Analysis

Determination of primary polar metabolites was performed as described by Lisec et al. [138] and Michailidis et al. [139] with slight modifications. Leaf lyophilized material (0.03−0.04 gr) from the best performing line under stress (*Pvgstu3–3*) and WT plants under control and combined DT stress treatment at 0 h and 9 days (three biological replicates). The leaf tissue was transferred in 2 mL tubes with the addition of 1400 μL precooled (−20 °C) pure methanol. An internal quantitative standard of adonitol (100 μL of 0.2 mg mL^−1^) was added, and solutions were incubated for 10 min at 70 °C, followed by a centrifugation step (11,000 g, 4 °C, 10 min). The collected supernatant was mixed with 750 μL chloroform (–20 °C) and 1500 μL dH_2_O (4 °C) and centrifuged at 2200 g, 4 °C, for 10 min. The upper polar phase (150 μL) was transferred into a 1.5 mL vial glass and placed under a vacuum until dried. The dried residues were redissolved in 40 μL of 20 mg mL^−1^ methoxyamine hydrochloride and incubated for 120 min at 37 °C, followed by treatment with 70 μL of N-methyl-N-(trimethylsilyl) trifluoroacetamide reagent (MSTFA) and incubation for 30 min at 37 °C. GC–MS analysis was carried out in Thermo Trace Ultra GC equipped with auto-sampler ISQ MS and TriPlus RSH (Switzerland). The mass range of m/z 550 was recorded after 5 min of solvent delay. The peak area integration and chromatogram visualization were performed using the X-calibers processing software. Standards were used for peak identification on the NIST11 database in case of unknown peaks [139]. The detected metabolites were assessed based on the relative response compared to the internal standard of adonitol and expressed as relative abundance.

### 4.6. Statistical Analysis

The statistical analysis for the morpho-physiological data was performed using the computing environment R-4.0.3 (R Core Team) [140]. The effects of stress treatments and the genotypes at harvest and treatments, genotypes and time (days-where applicable) on the morpho-physiological parameters were assessed using two-way and three-way ANOVA, respectively, with the ez and afex packages [141,142]. All data were tested for normality (Shapiro test), and if normality failed and transformations were attempted. Data were also tested with Mauchly’s test for sphericity, and if the assumption of sphericity was violated, the corresponding Greenhouse–Geisser corrections were performed. If significant differences were found among treatments, then Tukey’s HSD post hoc test was performed to determine specific treatment differences using the Agricolae package [143]. The analysis for the metabolites was conducted using the SPSS (IBM SPSS Statistics SPSS v24.0., Armonk, NY, USA) and metabolites, which presented statistically significant differences were based on Duncan’s multiple range test for the effect of genotype x treatment interaction and Student’s t-test for the single effects of treatment and genotype with significance level *p* ≤ 0.05 (Supplemental Tables S4 and S5). The raw data are presented in Table S6, and the relative response was adjusted by removing the background of the *Pvgstu3–3* and WT at 0 days (control) from the respective plants on day nine.

## 5. Conclusions

To the best of our knowledge, this is the first transcriptomics and metabolomics characterization of the protective effect of *Pvgstu* against abiotic stresses, contributing towards a better understanding of the mechanisms that govern stress-tolerance and, specifically, tolerance under combined and single drought and heat stresses, under the spectrum of climate change. It is an initiative towards understanding the in planta function of common bean *gstu* genes with validated catalytic function [21,33]. Taking into consideration the major negative impact of drought and heat stress to crop productivity, the results presented herein would help to elucidate the role of GST in plant stress-tolerance and provide further insights into the potential functional mechanism of GST towards productive agriculture in stress affected areas and enhancing crop safety and production.

## Figures and Tables

**Table 1:** Growth traits of the *Pvgstu* transgenic lines and WT tobacco plants grown for nine days in drought (D), heat (T) and a combination of drought and heat (DT) stresses. Tukey’s HSD (T_HSD_) post hoc test was performed for the treatment effect on shoot and root length, fresh matter (M_F_) and the number of leaves for each genotype. Data are means ± SE (WT: *n* = 4; *Pvgstu* lines: *n* = 5). Different letters indicate significant differences between treatments for each genotype at *p* < 0.05.

Mid-harvest (Day 9)									
Genotypes	Treatment	Shoot Length	T_HSD_	Root Length	T_HSD_	M_F_	T_HSD_	Number of Leaves	T_HSD_
*Pvgstu2–2.19*	C	12 ± 0.85	a	9.3 ± 1.4	a	6.46 ± 0.41	a	11 ± 0.71	a
	D	9.04 ± 0.77	ab	7.7 ± 0.62	ab	3.48 ± 0.3	b	8.6 ± 0.24	b
	T	9.66 ± 1.09	ab	6.9 ± 0.51	ab	5.74 ± 0.66	a	8.8 ± 0.37	b
	DT	8.2 ± 0.3	b	4.24 ± 0.708	b	1.14 ± 0.09	c	7.6 ± 0.24	b
*Pvgstu3–3.4*	C	11.28 ± 0.47	a	8.84 ± 0.88	a	6.3 ± 0.63	a	10 ± 0.55	a
	D	9 ± 0.524	a	8.88 ± 0.82	a	3.7 ± 0.28	b	9 ± 0.32	a
	T	9.98 ± 0.87	a	6.9 ± 1.67	a	6.04 ± 0.24	a	8.8 ± 0.66	a
	DT	9 ± 0.464	a	5.5 ± 0.55	a	1.08 ± 0.14	c	6.8 ± 0.37	b
WT	C	12.12 ± 1.01	a	13.62 ± 2.01	a	5.6 ± 0.196	a	12.5 ± 1.44	a
	D	10.12 ± 0.96	a	8.5 ± 1.67	ab	3.45 ± 0.35	b	10 ± 0.41	a
	T	10.87 ± 0.87	a	8 ± 1.17	ab	4.55 ± 0.3	a	9.75 ± 1.25	a
	DT	9.82 ± 0.64	a	6.07 ± 0.47	b	0.65 ± 0.08	c	5 ± 0	b

**Table 2:** Growth traits of *Pvgstu* transgenic lines and WT tobacco plants grown for 16 days in drought (D), heat (T), and combination of drought and heat (DT) stresses and their recovery measured on day 16. Tukey’s HSD (T_HSD_) post hoc test was performed for the interaction effect of genotype × treatment on shoot and root length, fresh weight (M_F_) and the number of leaves. Data are the mean ± SE (WT: *n* = 4; *Pvgstu* lines: *n* = 7). Different letters indicate significant differences between genotypes for each treatment at *p* < 0.05.

Final Harvest (Day 16)									
Treatment	Genotype	Shoot Length	T_HSD_	Root Length	T_HSD_	M_F_	T_HSD_	Number of Leaves	T_HSD_
C	*Pvgstu2–2.19*	15.08 ± 0.64	a	9.94 ± 0.77	a	8.04 ± 0.5	ab	27.45 ± 1.97	a
	*Pvgstu3–3.4*	14.24 ± 0.846	a	10.23 ± 0.77	a	9.28 ± 0.53	a	22.33 ± 0.84	b
	WT	12.02 ± 0.72	a	13.2 ± 1.51	a	6.8 ± 0.66	b	21.75 ± 2.17	b
D	*Pvgstu2–2.19*	9.89 ± 0.52	a	6.88 ± 0.64	a	2.46 ± 0.18	a	23.1 ± 1.24	a
	*Pvgstu3–3.4*	9.37 ± 0.37	a	6.01 ± 0.44	a	2.5 ± 0.17	a	22.7 ± 1.38	a
	WT	10.85 ± 0.6	a	7.07 ± 0.83	a	2.17 ± 0.25	a	23.75 ± 1.11	a
T	*Pvgstu2–2.19*	10.5 ± 0.52	a	5.09 ± 0.144	b	5.6 ± 0.36	b	26 ± 1.64	a
	*Pvgstu3–3.4*	12.02 ± 0.52	a	6.65 ± 0.54	a	8.05 ± 0.42	a	27.69 ± 1.1	a
	WT	10.45 ± 0.66	a	5.97 ± 0.49	ab	5.85 ± 0.36	b	24 ± 1.47	a
DT	*Pvgstu2–2.19*	7.82 ± 0.7	a	3.28 ± 0.38	b	0.46 ± 0.05	ab	15.62 ± 1.33	a
	*Pvgstu3–3.4*	9.76 ± 0.58	a	5.06 ± 0.82	ab	0.64 ± 0.048	a	10.43 ± 1.91	ab
	WT	9.82 ± 0.65	a	6.07 ± 0.47	a	0.65 ± 0.086	a	5 ± 0	b
Recovery	*Pvgstu2–2.19*	10.42 ± 0.73	ab	4.82 ± 0.28	a	3.64 ± 0.14	b	23.4 ± 1.5	a
	*Pvgstu3–3.4*	8.8 ± 0.57	b	5.96 ± 0.41	a	5.8 ± 0.49	a	27.3 ± 3.35	a
	WT	11.72 ± 0.64	a	5.36 ± 0.92	a	3.12 ± 0.47	b	26.5 ± 2.22	a

**Table 3:** Annotation and DEGs (total number and percentages) of pathways (results were determined using KEGG) showing a representative list of the significantly enriched biological process of a) WT vs. *Pvgstu3–3.4* in stress-free control (C) conditions (0 h) (groups 1 vs. 2, respectively); b) stress-free *Pvgstu3–3.4* (C) vs. *Pvgstu3–3.4* at 48 h under DT (groups 2 vs. 4, respectively); c) stress-free WT (C) vs. *Pvgstu3–3.4* at 8 h under DT (groups 1 vs. 3, respectively).; d) stress-free WT (C) vs. *Pvgstu3–3.4* at 48 h under DT (groups 1 vs. 4, respectively). DT indicates combined drought and heat stresses.

Genotype/Treatment	Pathway Annotation	DEGs		Genes	
		Total Number (566)	%	Total Number (21,512)	%
**WT vs. *Pvgstu3–3.4*** **under stress-free conditions (C)**	Metabolic pathways	139	24.56%	3944	18.33%
	MAPK signaling pathway	34	6.01%	768	3.57%
	NF-kappa B signaling pathway	31	5.48%	528	2.45%
	Arachidonic acid metabolism	14	2.47%	142	0.66%
	Tryptophan metabolism	14	2.47%	201	0.93%
	Peroxisome	14	2.47%	297	1.38%
	Retinol metabolism	12	2.12%	181	0.84%
	Glycerophospholipid metabolism	11	1.94%	172	0.80%
	Linoleic acid metabolism	10	1.77%	89	0.41%
	Amino sugar and nucleotide sugar metabolism	10	1.77%	162	0.75%
	Glycerolipid metabolism	9	1.59%	123	0.57%
	Metabolism of xenobiotics by cytochrome P450	9	1.59%	138	0.64%
	Steroid hormone biosynthesis	8	1.41%	98	0.46%
	Phospholipase D signaling pathway	8	1.41%	131	0.61%
	Phenylalanine metabolism	6	1.06%	91	0.42%
		**Total Number (668)**	**%**	**Total Number (21,512)**	**%**
**Stress-free WT (C) vs. *Pvgstu3–3.4* under 8 h (DT)**	Metabolic pathways	174	26.05%	3944	18.33%
	Starch and sucrose metabolism	20	2.99%	282	1.31%
	amino sugar and nucleotide sugar metabolism	18	2.69%	162	0.75%
	Galactose metabolism	14	2.10%	136	0.63%
	Arachidonic acid metabolism	14	2.10%	142	0.66%
	Retinol metabolism	14	2.10%	181	0.84%
	Metabolism of xenobiotics by cytochrome P450	13	1.95%	138	0.64%
	Tryptophan metabolism	13	1.95%	201	0.93%
	Linoleic acid metabolism	9	1.35%	89	0.41%
	Glycine, serine and threonine metabolism	8	1.20%	91	0.42%
	Steroid hormone biosynthesis	8	1.20%	98	0.46%
		**Total Number (1588)**	**%**	**Total Number (21,512)**	**%**
**Stress-free WT (C) vs. *Pvgstu3–3.4* under 48 h (DT)**	Metabolic pathways	395	24.87%	3944	18.33%
	Carbon metabolism	55	3.46%	562	2.61%
	Biosynthesis of amino acids	40	2.52%	374	1.74%
	Starch and sucrose metabolism	39	2.46%	282	1.31%
	Tryptophan metabolism	28	1.76%	201	0.93%
	Arachidonic acid metabolism	27	1.70%	142	0.66%
	Amino sugar and nucleotide sugar metabolism	26	1.64%	162	0.75%
	Retinol metabolism	26	1.64%	181	0.84%
	Metabolism of xenobiotics by cytochrome P450	24	1.51%	138	0.64%
	Glycolysis/gluconeogenesis	24	1.51%	208	0.97%
	Glyoxylate and dicarboxylate metabolism	24	1.51%	224	1.04%
	Galactose metabolism	20	1.26%	136	0.63%
	Folate biosynthesis	17	1.07%	133	0.62%
	RNA polymerase	16	1.01%	116	0.54%
	Phospholipase D signaling pathway	16	1.01%	131	0.61%
		**Total Number (3967)**	**%**	**Total Number (21,512)**	**%**
**Stress-free *Pvgstu3–3.4* (C) vs. *Pvgstu3–3.4* at 48 h (DT)**	Metabolic pathways	853	21.50%	3944	18.33%
	Carbon metabolism	136	3.43%	562	2.61%
	Endocytosis	97	2.45%	443	2.06%
	Biosynthesis of amino acids	86	2.17%	374	1.74%
	ABC transporters	84	2.12%	285	1.32%
	Apoptosis	84	2.12%	379	1.76%
	Peroxisome	75	1.89%	297	1.38%
	Pyruvate metabolism	71	1.79%	311	1.45%
	Starch and sucrose metabolism	69	1.74%	282	1.31%
	Necroptosis	64	1.61%	226	1.05%
	Glycolysis/gluconeogenesis	55	1.39%	208	0.97%
	Glyoxylate and dicarboxylate metabolism	55	1.39%	224	1.04%
	Citrate cycle (TCA cycle)	55	1.39%	241	1.12%
	RNA degradation	53	1.34%	231	1.07%
	Drug metabolism—other enzymes	51	1.29%	183	0.85%
	Base excision repair	50	1.26%	195	0.91%
	Tryptophan metabolism	49	1.24%	201	0.93%
	Arachidonic acid metabolism	47	1.18%	142	0.66%
	Retinol metabolism	43	1.08%	181	0.84%
	Metabolism of xenobiotics by cytochrome P450	40	1.01%	138	0.64%

**Table 4:** Top 15 representative transcription factors (TFs) and the number of up- and downregulated genes in response to stress-free (C) conditions and combined drought and heat stress (DT) at 48 h in WT and *Pvgstu3–3.4* transgenic plants.

TFs	WT (C)/*Pvgstu3–3.4* (C)		WT (C)/*Pvgstu3–3.4* (DT) 48 h		*Pvgstu3–3.4* (C)/(DT) 48 h	
	No. of Up- Regulated	No. of Down- Regulated	No. of Up- Regulated	No. of Down- Regulated	No. of Up- Regulated	No. of Down- Regulated
**MYB**	27	13	66	33	87	56
**mTERF**	-	2	16	-	79	4
**MYB-related**	13	12	47	30	70	40
**FAR1**	3	-	25	-	51	4
**MADS**	1	2	13	5	37	11
**AP2-EREBP**	53	49	29	86	32	128
**NAC**	12	7	22	24	31	30
**Trihelix**	-	-	21	2	28	4
**ARF**	1	1	7	6	23	17
**HSF**	3	10	15	13	18	27
**ARR-B**	-	-	2	-	13	-
**WRKY**	21	32	12	54	8	71
**bZIP**	-	4	9	6	8	5
**SBP**	-	-	2	7	8	7
**Sigma70-like**	-	-	5	-	8	-

**Table 5:** Metabolites that were significantly (*p* < 0.05) altered in the different genotypes under combined drought and heat stress and control conditions. Positive values indicate fold-increase, whereas negative values fold-decrease.

*Pvgstu3–3.4* (DT)/WT (DT)		*Pvgstu3–3.4* (C)/WT (C)	
Metabolites	Log_2_-Fold Change	Metabolites	Log_2_-Fold Change
Arginine	20.5	Glycerol	2.2
Fructose	5.3	Quinic acid	1.2
Glucose	4.0	Ethanolamine	0.7
Glycine	3.2	Sucrose	−0.4
Pantothenate	3.1	Fructose	−0.6
Myoinisitol	2.9	Glucose	−0.8
Threonic acid	2.3	Myoinisitol	−0.8
Tyrosine	2.2	Glutamic acid	−0.8
Sorbitol	2.1	Nicotine	−0.9
Phenylethanolamine	1.8	Malic acid	−0.9
Sucrose	1.6	beta_Alanine	−0.9
Xylose	1.4	Isoleucine	−0.9
Proline	1.3	GABA	−1.0
Threonine	0.6	Glycine	−1.0
Arabinose	−0.3	Arabinose	−1.0
Meso_eryrthitol	−0.3	Proline	−1.0
Glycerol	−0.5	Sorbitol	−1.0
Phenylalanine	−0.5	Glyceric acid	−1.0
Ethanolamine	−0.5	Valine	−1.0
Serine	−0.6	Phenylalanine	−1.0
Glutamine	−0.7	Threose	−1.0
Alanine	−0.9	Oxoproline	−1.0
beta_Alanine	−0.9	Mannitol	−1.0
Lysine	−0.9		
GABA	−0.9		
Galactose	−1.0		
Tryptophan	−1.0		

**Table 6:** Plant material used for RNA extraction and transcriptomics analysis with of the WT and *Pvgstu3–3.4* transgenic line with 2 and 6 biological replicates, respectively, under stress-free and DT combined stress conditions at different time points.

Genotype	Treatment	Analysis group
WT	Control; 0 h	Group 1
*Pvgstu3–3.4*	Control; 0 h	Group 2
	DT combined stress; 8 h	Group 3
	DT combined stress; 48 h	Group 4

## Data Availability

The data presented in this study are available in the article or supplementary material.

## Supplementary Materials

The following are available online at https://www.mdpi.com/1422-0067/22/5/2352/s1.
