# Supplementary material for: Overexpression of A Biotic Stress-Inducible Pvgstu Gene Activates Early Protective Responses in Tobacco under Combined Heat and Drought
Source: Int J Mol Sci. 2021 Feb 26;22(5):2352. doi: 10.3390/ijms22052352 (PMC7956764; doi:10.3390/ijms22052352)
Supplement: Supplementary file 1 [file ijms-22-02352-s001.zip › ijms-1090292-sup/Supplementary material.docx]

**Supplemental Results**

**Evaluation of the transgenes**

Double digestion and ligation were performed to generate the recombinant plasmids pART27-*Pvgstu3-3* and pART27-*Pvgstu2-2* and were verified by PCR. The amplified DNA fragment was 795 bp for the *Pvgstu2-2* gene and 786 bp for the *Pvgstu3-3* gene. No bands were observed for the pART27 vector amplified regions cut with the restriction enzymes EcoRI-HindIII and EcoRI-XbaI that were used as negative controls (Figure S1a). Following the genetic transformation of the *E. coli* cells with the pART27-*Pvgstu2-2* and pART27-*Pvgstu*3–3, 5 colonies for each recombinant plasmid were screened for successful transformation. Only two out of the five colonies having the pART27-*Pvgstu2-2* were positively transformed having a 795 bp band, whereas all five colonies were positive for the pART27-*Pvgstu3-3* having a 786 bp band (Figure S1b). The positive colonies were further tested by restriction enzyme analysis and digestion of the transformed pART27-*Pvgstu2-2* colonies with the restriction enzymes EcoRI and BamHI were expected to yield 4 bands of 12535 bp, 1276 bp, 502 bp and 188 bp, as it was observed in only one clone. Digestion of pART27-*Pvgstu3-3* with HindIII was expected to yield 2 bands of 14.224 bp and 246 bp as shown in the 2 positive clones (Figure S1c). Digestion of pART27-*gstu*4 with the restriction enzymes EcoRI and BamHI gave three bands 12535 bp, 1276 bp, and 693 bp and digestion with the restriction enzyme HindIII a linear molecule of pART27-*gstu*4. This different zone pattern between pART27-*gstu*4 and the plasmids pART27-*Pvgstu*2-2 or pART27-*Pvgstu3-3* confirmed the successful transformation of the colonies with these plasmids and not with pART27-*gstu*4 vector (Figure S1c). After, sequencing, one of the selected clones for each recombinant plasmid was verified as positive.


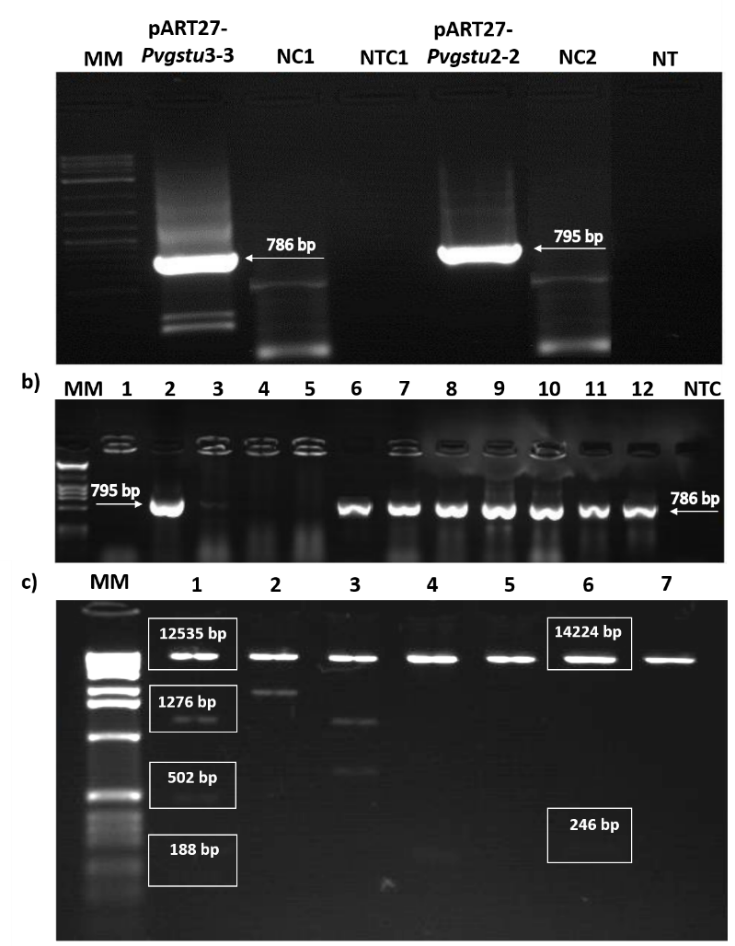


**Figure S1:** PCR products for plasmid constructs and *E. coli* transformation: **a)** assessment of recombinant plasmids after ligase reactions, pART27-*Pvgstu3*-3: recombinant plasmid pART27-*Pvgstu3*-3, NC1: recombinant pART27 plasmid vector digested with EcoRI and Xba restriction enzymes (negative control 1), pART27-*Pvgstu2-2*: recombinant plasmid pART27-*Pvgstu*2-2, NC2: PART27 plasmid vector digested with EcoRI and HindIII restriction enzymes (negative control 2). **b)** PCR-verification of *E. coli* cell transformation with recombinant plasmids pART27-*Pvgstu*2-2 and pART27-*Pvgstu*3-3. Lanes 1-5: PCR products from 5 colonies of potentially transformed *E. coli* cells with plasmid pART27-*Pvgstu2-2*. Lane 6: recombinant plasmid pART27-*Pvgstu*2-2 (positive control). Lanes 7-11: PCR products from 5 colonies of potentially transformed *E. coli* cells with plasmid pART27- *Pvgstu*3-3, and Lane 12: Recombinant plasmid pART27-*Pvgstu*3-3 (positive control). **c)** products of the restriction enzyme reactions for the verification of recombinant plasmids *Pvgstu*2-2 and pART27-*Pvgstu*3-3. Lanes 1 and 2: clones of pART27- *Pvgstu2-2*. Lane 3: recombinant plasmid pART27-*gstu*4. Lanes 4-6: clones pART27-*Pvgstu*3-3. Lane 7: recombinant plasmid pART27-*gstu*4. Lanes MM: 1Kb molecular marker and NTC: DNA-free template (no template control).

**
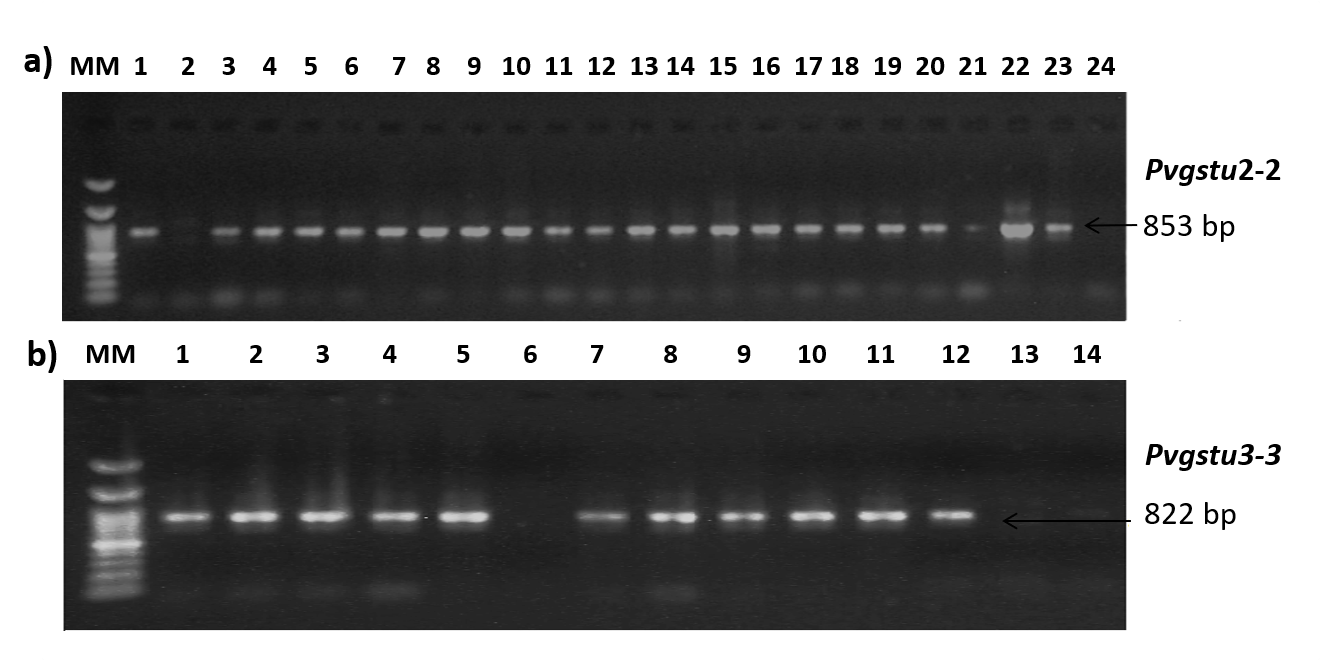
Figure S2:** Gel electrophoresis (1% agarose) of the RT-PCR products of independently transformed transgenic tobacco lines. *Pvgstu*2-2 with: [1-21] Putative genetically transformed plants with the *Pvgstu*2-2 gene; [22] Recombinant plasmid pART27-*Pvgstu*2-2 (positive control); [23] Positively identified genetically transformed plant with the *Gmgstu4* gene (positive control); [24] Non-genetically transformed *N. tabacum* plant (negative control-WT); [25]: Sample free of DNA template. *Pvgstu*3-3 with: [1-10] Putative genetically transformed plants with the gene *Pvgstu*3-3, [11] Recombinant plasmid pART27- *Pvgstu*3-3 (positive control); [12] Identified genetically transformed plant with the *Pvgstu*2-2 gene (positive control); [13] Non-genetically transformed *N. tabacum* plant (negative control-WT) and [14] Sample free of DNA template. MM: molecular marker 100 bp. Lanes with bands at 853 bp and 822 bp fragments indicate individual plants that contain the *Pvgstu*2-2 and *Pvgstu*3-3 genes respectively.


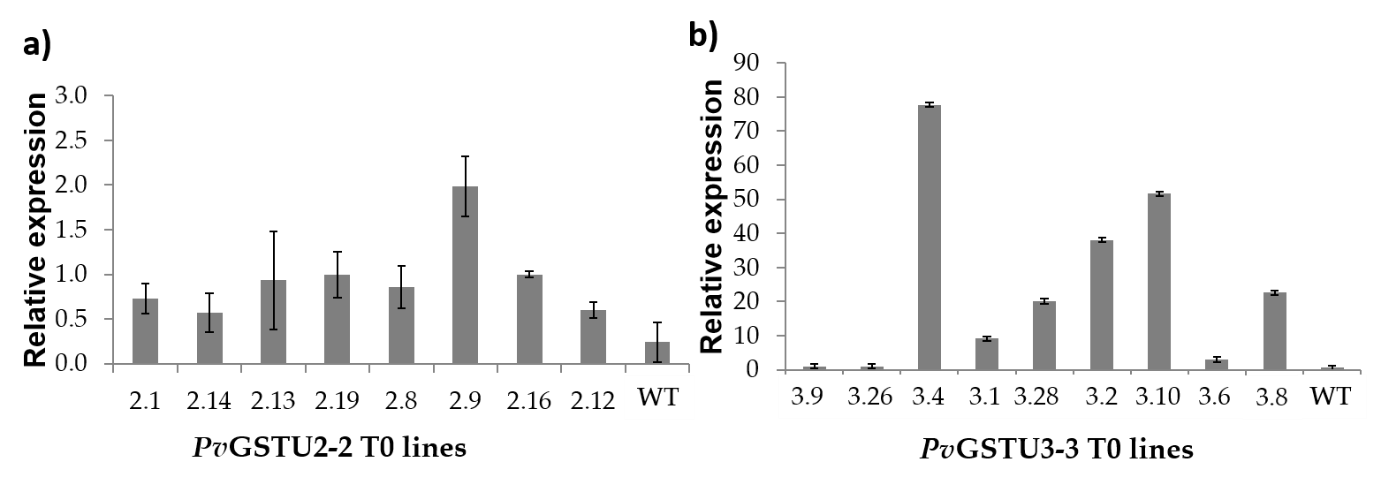
**Figure S3:** Screening of transgenic tobacco lines through relative expression analysis of the a) *Pvgstu*2-2 and b) *Pvgstu*3-3 transgenes.

**Table S1**: Mean value (m) and standard deviation (sd) of the CT (threshold cycle) for the relative gene expression of *Pvgstu*2-2 and *Pvgstu*3-3 genes using *β-actin* as internal reference control, and the resulting relative quantitative expression (2^-ΔΔCT^ ± sd, n=3) for each of the T0 genetically transformed *N. tabacum* lines.

| **Τ0 lines** | ***m*±*sd* CT *Pvgstu*** | ***m*±*sd* CT *β-actin*** | **2^-ΔΔCT^±sd** |
| --- | --- | --- | --- |
| ***Pvgstu*2-2.1** | 11.11 ± 0.07 | 14.75 ± 0.16 | 0.73 ± 0.17 |
| ***Pvgstu2-2.14*** | 12.70 ± 0.08 | 15.99 ± 0.21 | 0.57 ± 0.22 |
| ***Pvgstu*2-2.13** | 11.64 ± 0.34 | 15.64 ± 0.42 | 0.93 ± 0.55 |
| ***Pvgstu*2-2.19** | 11.18 ± 0.06 | 15.27 ±0.25 | 0.99 ± 0.25 |
| ***Pvgstu*2-2.8** | 11.99 ± 0.08 | 15.87 ±0.23 | 0.86 ± 0.24 |
| ***Pvgstu*2-2.9** | 10.98 ± 0.08 | 16.07 ± 0.33 | 1.99 ± 0.33 |
| ***Pvgstu*2-2.16** | 12.92 ± 0.03 | 17.02 ± 0.03 | 1.00 ± 0.04 |
| ***Pvgstu*2-2.12** | 12.37 ±0.02 | 15.45 ±0.08 | 0.60 ± 0.08 |
| ***Pvgstu3-3.9*** | 15.55±0.41 | 15.18±0.65 | 1.00±0.77 |
| ***Pvgstu3-3.26*** | 17.67±0.05 | 17.3±1.41 | 1.00±1.41 |
| ***Pvgstu3-3.4*** | 13.59±0.47 | 19.5±0.19 | 77.71±0.51 |
| ***Pvgstu3-3.1*** | 15.78±0.19 | 18.6±0.53 | 9.13±0.56 |
| ***Pvgstu3-3.28*** | 14.08±0.14 | 18.04±0.73 | 20.11±0.74 |
| ***Pvgstu3-3.2*** | 12.86±0.09 | 17.74±0.39 | 38.05±0.40 |
| ***Pvgstu3-3.10*** | 12.51±0.36 | 17.83±0.3 | 51.62±0.47 |
| ***Pvgstu3-3.6*** | 15.44±0.45 | 16.67±0.49 | 3.03±0.66 |
| ***Pvgstu3-3.8*** | 15.09±0.54 | 19.22±0.29 | 22.63±0.61 |
| **Wild type (WT)** | - | 20.24±0.37 | - |


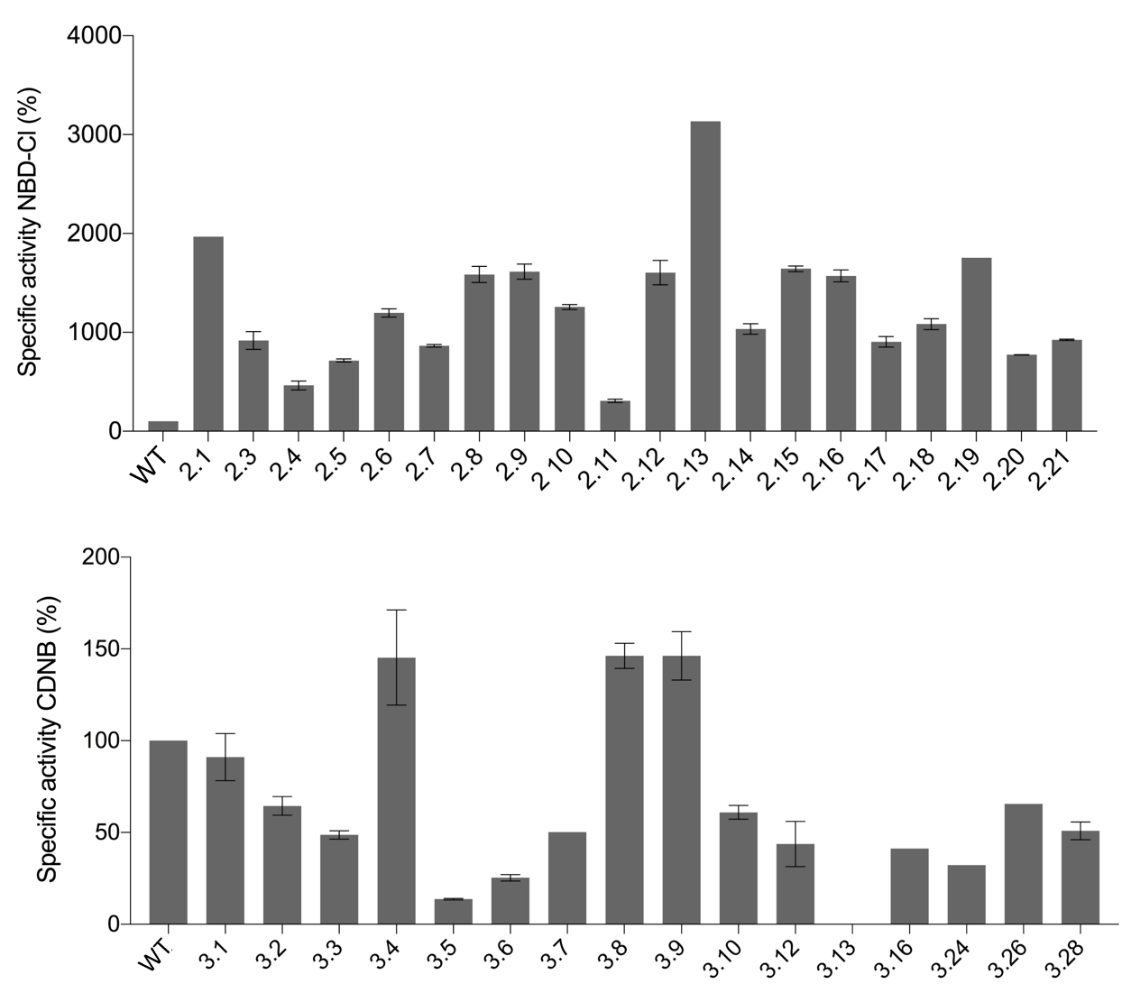


**Figure S4:** Enzymatic activity for a) *Pvgstu*2-2 towards the NBD-CI substrate and b) *Pvgstu*3-3 towards the CDNB substrate.


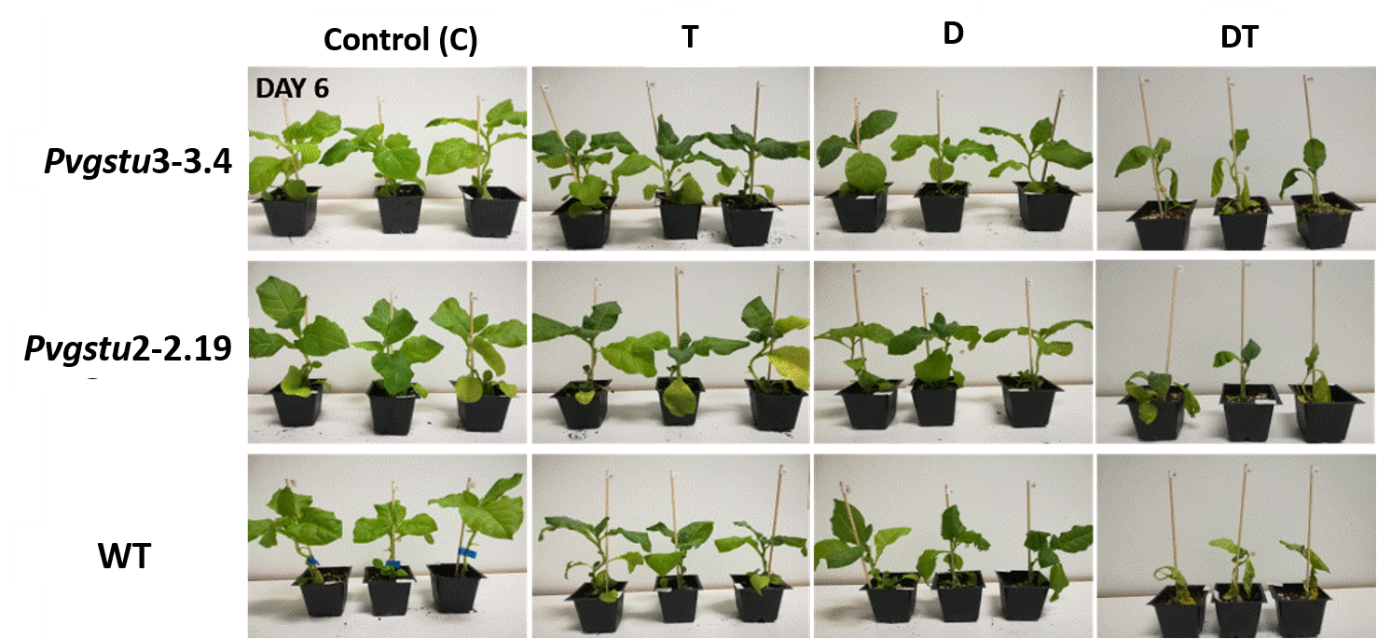


**Figure S5:** Effect heat (T), drought (D) and combination of drought and heat (DT) stress on growth of transgenic lines (*Pvgstu*3-3.4 and *Pvgstu*2-2.19) and WT tobacco plants after 6 days compared to the control (C) conditions.

**Table S2:** Growth traits of *Pvgstu* transgenic lines (*Pvgstu*3-3.4 and *Pvgstu*2-2.19) and the wild type (WT) tobacco plants grown for 16 days in drought (D), heat (T) and combination of drought and heat (DT) stresses and their recovery measured on day 16. Tukey HSD (T_HSD_) post-hoc test was performed for the interaction effect of genotype × treatment on shoot and root length, fresh weight (M_F_) and the number of leaves. Data are the mean ± SE (WT: n=4; *Pvgstu* lines: n=7). Different letters indicate significant differences between treatments for each genotype at p<0.05.

| **Day 16 (Harvest)** | | | | | | | | | |
| --- | --- | --- | --- | --- | --- | --- | --- | --- | --- |
| **Genotypes** | **Treatments** | **Shoot length** | **T_HSD_** | **Root length** | **T_HSD_** | **M_F_** | **T_HSD_** | **Leaves** | **T_HSD_** |
| ***Pvgstu***2-2.19 | C | 14.24±0.64 | a | 9.94±0.77 | a | 8.04±0.505 | a | 27.4±1.96 | a |
|  | T | 10.51±0.52 | b | 5.09±0.144 | bc | 5.6±0.367 | b | 26±1.64 | a |
|  | D | 9.89±0.52 | bc | 6.88±0.636 | b | 2.46±0.181 | c | 23.1±1.24 | ab |
|  | DT | 7.8±0.73 | c | 3.28±0.38 | c | 0.46±0.053 | d | 15.6±1.33 | b |
|  | Recovery | 10.42±0.45 | bc | 4.82±0.28 | bc | 3.64±0.143 | c | 23.4±1.5 | ab |
| ***Pvgstu***3-3.4 | C | 15.1±0.85 | a | 10.23±0.77 | a | 9.28±0.529 | a | 22.33±0.83 | a |
|  | T | 12.02±0.52 | b | 6.65±0.54 | b | 8.05±0.419 | a | 27.69±1.1 | a |
|  | D | 9.4±0.37 | c | 6.015±0.44 | b | 2.5±0.172 | c | 22.76±1.38 | a |
|  | DT | 9.75±0.58 | bc | 5.057±0.816 | b | 0.64±0.048 | d | 10.43±1.91 | b |
|  | Recovery | 8.8±0.28 | c | 5.966±0.409 | b | 5.8±0.491 | b | 27.3±3.35 | a |
| **WT** | C | 12.025±0.72 | a | 13.2±1.51 | a | 6.8±0.663 | a | 21.75±2.17 | a |
|  | T | 10.45±0.66 | a | 5.975±0.49 | b | 5.85±0.36 | a | 24±1.47 | a |
|  | D | 10.85±0.61 | a | 7.075±0.83 | b | 2.175±0.25 | b | 23.75±1.11 | a |
|  | DT | 9.825±0.64 | a | 6.075±0.47 | b | 0.65±0.086 | c | 5±0 | b |
|  | Recovery | 11.725±0.82 | a | 5.366±0.92 | b | 3.125±0.47 | b | 26.5±2.21 | a |

**Table S3**: Physiological responses of the transgenic lines *Pvgstu*3-3.4 and *Pvgstu*2-2.19 and the wild type (WT) plants to control (C), drought (D), heat (T) and combination of drought and heat stresses (DT), at nine and 16 days. Tukey HSD (T_HSD_) post-hoc test was performed for the interaction effect genotype × treatment for each time point on the maximum quantum yield of PSII (*F*v/*F*m) and relative chlorophyll content (CCI). No values could be obtained from leaves at DT treatment after 16 days. Data are mean ± Standard Error (WT: n=4; *Pvgstu* lines at 9 days: n=5; at 16 days: n=7). Different letters indicate significant differences between treatments for each genotype at p<0.05.

| **Genotypes** | **Treatments** | **Days** | ***F*v*F*m** | **T_HSD_** | **Chl** | **T_HSD_** |
| --- | --- | --- | --- | --- | --- | --- |
| *Pvgstu*2-2.19 | C | 9 | 0.8±0.002 | a | 7.08±0.38 | a |
|  | T | 9 | 0.81±0.003 | a | 9.52±1.09 | a |
|  | D | 9 | 0.81±0.0013 | a | 11.12±1.079 | a |
|  | DT | 9 | 0.77±0.01 | b | 8.54±1.368 | a |
| *Pvgstu*3-3.4 | C | 9 | 0.8±0.002 | b | 8.2±0.79 | b |
|  | T | 9 | 0.82±0.004 | a | 14.18±2.25 | a |
|  | D | 9 | 0.817±0.002 | a | 11±1.26 | ab |
|  | DT | 9 | 0.759±0.003 | c | 9.4±0.94 | ab |
| WT | C | 9 | 0.796±0.0068 | a | 9.925±0.558 | a |
|  | T | 9 | 0.82±0.0034 | a | 14.05±0.27 | a |
|  | D | 9 | 0.78±0.0091 | a | 11.35±0.24 | a |
|  | DT | 9 | 0.74±0.013 | b | 10.875±2.87 | a |
| *Pvgstu*2-2.19 | C | 16 | 0.8±0.005 | a | 7.94±0.438 | c |
|  | T | 16 | 0.81±0.002 | a | 25.9±1.625 | a |
|  | D | 16 | 0.8±0.0069 | a | 19.26±1.948 | b |
| *Pvgstu*3-3.4 | C | 16 | 0.81±0.0035 | a | 7.05±0.294 | b |
|  | T | 16 | 0.803±0.011 | a | 21.58±2.358 | a |
|  | D | 16 | 0.789±0.0068 | a | 16.08±2.047 | a |
| WT | C | 16 | 0.794±0.005 | a | 7.55±0.805 | b |
|  | T | 16 | 0.78±0.012 | a | 13.75±2.87 | ab |
|  | D | 16 | 0.751±0.014 | a | 18.7±4.39 | a |

**Table S4**: Physiological responses of the transgenic lines *Pvgstu*3-3.4 and *Pvgstu*2-2.19 and the wild type (WT) plants to combination of drought and heat stresses (DT) and recovery phase, between days 10 (due to significant wilting) and 16 (when the recovery was assessed after re-watering on day 9), respectively. No values could be obtained from leaves under DT treatment at 16 days. Tukey HSD (T_HSD_) post-hoc test was performed for the interaction effect genotype × treatment on the maximum quantum yield of PSII (*F*v/*F*m) and relative chlorophyll content (CCI). Data are mean ± Standard Error (WT: n=4; *Pvgstu* lines n=5). Different letters indicate significant differences between treatments for each genotype at p<0.05.

| **DT *versus* Recovery phase** | | | | | |
| --- | --- | --- | --- | --- | --- |
| **Genotypes** | **Treatments** | **Chl** | **T_HSD_** | ***F*v*F*m** | **T_HSD_** |
| *Pvgstu*2-2.19 | DT | 5.55±0.45 | b | 5.55±0.45 | b |
|  | Recovery | 23.14±3.64 | a | 23.14±3.64 | a |
| *Pvgstu*3-3.4 | DT | 7.125±1.07 | b | 7.125±1.07 | b |
|  | Recovery | 28.66±2.21 | a | 28.66±2.21 | a |
| WT | DT | 5.375±0.77 | b | 5.375±0.77 | b |
|  | Recovery | 24.32±4.84 | a | 27.175±5.04 | a |

**
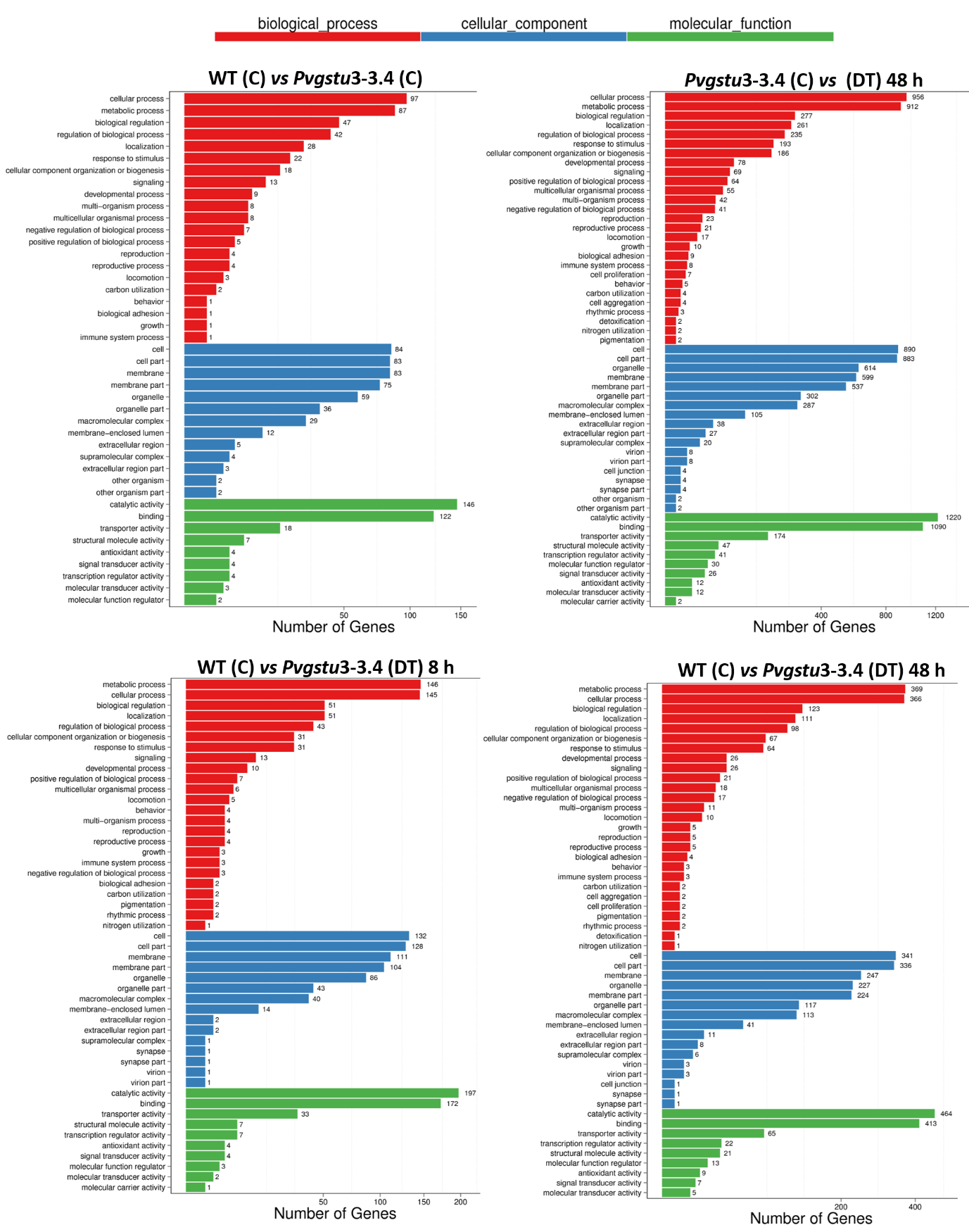
**

**Figure S6:** Gene Ontology (GO) functional classification of DEGs: a) WT *vs* *Pvgstu*3-3.4 in non-stressed control (C) conditions [0 h] (groups 1 *vs* 2 respectively); b) non-stressed *Pvgstu*3-3.4 (C) *vs Pvgstu*3-3.4 at 48h under D_T (groups 2 vs 4, respectively); c) non-stressed WT (C) *vs Pvgstu*3-3.4 at 8h under D_T (groups 1 *vs* 3, respectively).; d) non-stressed WT (C) *vs* *Pvgstu*3-3.4 at 48h under D_T (groups 1 *vs* 4, respectively). D_T indicates combined drought and heat stresses. Unigenes were assigned to three main categories: biological process, cellular components, and molecular function.

**Table S5**: Primary metabolites of *Pvgstu*3-3.4 and WT plants under combined D_T stress (9 days) compared to control conditions. Mean values of three independent determinations for each treatment were expressed as relative abundance compared to internal standard adonitol and are reported as background-free of the control conditions at 0 days. Significant differences between genotypes for each treatment are presented with asterisk (*) at p<0.05.

| **Metabolites** | **Genotype** | | | | | | **Treatment** | | | | | |
| --- | --- | --- | --- | --- | --- | --- | --- | --- | --- | --- | --- | --- |
|  | ***Pv*GST**3-3.4 | | | **WT** | | | **Control** | | | **DT** | | |
|  | **Mean** |  | **±SE** | **Mean** |  | **±SE** | **Mean** |  | **±SE** | **Mean** |  | **±SE** |
| **Soluble sugars** | | | | | | | | | | | | |
| **Xylose** | **4.561** | ***** | 2.973 | 2.059 |  | 1.218 | 0.812 |  | 0.175 | **5.808** | ***** | 2.163 |
| **Arabinose** | 1.846 |  | 1.382 | **3.559** | ***** | 1.525 | 0.857 |  | 0.657 | **4.548** | ***** | 0.773 |
| **Threose** | 12.229 |  | 9.344 | **67.941** | ***** | 25.198 | 23.413 |  | 17.632 | **56.757** | ***** | 31.765 |
| **Fructose** | **28.785** | ***** | 22.176 | 4.899 |  | 3.261 | 0.521 |  | 0.199 | **33.164** | ***** | 18.812 |
| **Glucose** | **40.83** | ***** | 31.374 | 8.819 |  | 5.855 | 0.924 |  | 0.474 | **48.725** | ***** | 25.304 |
| **Galactose** | 14.726 |  | 11.503 | **352.931** | ***** | 282.74 | 0.511 |  | 0.379 | **367.15** | ***** | 272.14 |
| Erythrose | 15.813 |  | 12.881 | 7.568 |  | 5.954 | 1 |  | 0 | **22.381** | ***** | 9.19 |
| **Mannose-6-deoxy** | **1842.36** | ***** | 1442.03 | 605.121 |  | 535.41 | 1 |  | 0 | **2446.5** | ***** | 1015.48 |
| Sucrose | **2.018** | ***** | 0.569 | 1.583 |  | 0.462 | 1.722 |  | 0.368 | 1.879 |  | 0.672 |
| **Total** | **7.589** | ***** | 5.005 | 2.507 |  | 0.542 | 1.53 |  | 0.342 | **8.565** | ***** | 4.258 |
| **Soluble alcohols** | | | | | | | | | | | | |
| Glycerol | 0.804 |  | 0.173 | 0.765 |  | 0.374 | 0.618 |  | 0.289 | **0.951** | ***** | 0.23 |
| **Meso_eryrthitol** | 1.889 |  | 1.483 | **4.963** | ***** | 1.327 | 2.137 |  | 2.007 | **4.715** | ***** | 0.879 |
| Mannitol | 15.665 |  | 12.164 | 15.593 |  | 4.988 | 5.918 |  | 4.711 | **25.34** | ***** | 6.123 |
| **Sorbitol** | **146.96** | ***** | 119.97 | 63.246 |  | 29.59 | 16.652 |  | 12.568 | **193.55** | ***** | 88.46 |
| Myoinisitol | 3.387 |  | 1.953 | 3.485 |  | 1.658 | 3.225 |  | 1.843 | 3.647 |  | 1.765 |
| **Total** | 3.336 |  | 1.914 | 3.191 |  | 1.357 | 2.88 |  | 1.577 | 3.646 |  | 1.688 |
| **Organic acids** | | | | | | | | | | | | |
| Glyceric acid | 7.249 |  | 6.357 | 2.859 |  | 0.964 | 1.101 |  | 0.871 | **9.007** | ***** | 5.254 |
| Malic acid | 10.492 |  | 6.094 | **33.428** | ***** | 9.139 | 17.999 |  | 12.308 | 25.921 |  | 10.398 |
| **Threonic acid** | **11.059** | ***** | 6.901 | 4.779 |  | 1.134 | 3.171 |  | 0.631 | **12.666** | ***** | 5.769 |
| **2-Oxoglutaric acid** | **91.288** | ***** | 85.519 | 16.098 |  | 12.985 | 0.599 |  | 0.31 | **106.78** | ***** | 76.127 |
| Citric acid | 27.73 |  | 32.517 | 24.086 |  | 18.158 | 1 |  | 0 | **50.816** | ***** | 25.428 |
| Quinic acid | **1.871** | ***** | 0.569 | 0.955 |  | 0.188 | 1.51 |  | 0.459 | 1.317 |  | 0.624 |
| **Gluconic acid** | **2.387** | ***** | 2.021 | 0.713 |  | 0.593 | 0.047 |  | 0.006 | **3.054** | ***** | 1.601 |
| 2-Aminoadipic acid | 21.666 |  | 18.493 | 25.006 |  | 20.975 | 1 |  | 0 | **45.672** | ***** | 13.659 |
| **Total** | 6.533 |  | 3.734 | 6.17 |  | 1.54 | 3.623 |  | 1.427 | **9.08** | ***** | 2.32 |
| **Amino acids** | | | | | | | | | | | | |
| **Alanine** | 0.567 |  | 0.105 | **2.071** | ***** | 1.075 | 0.801 |  | 0.282 | **1.837** | ***** | 1.202 |
| **Valine** | 4.513 |  | 3.505 | **6.616** | ***** | 4.888 | 0.282 |  | 0.213 | **10.847** | ***** | 1.996 |
| Isoleucine | 3.479 |  | 2.754 | 2.831 |  | 1.895 | 0.226 |  | 0.201 | **6.085** | ***** | 0.982 |
| **Proline** | **147.107** | ***** | 115.108 | 63.636 |  | 50.132 | 0.262 |  | 0.195 | **210.48** | ***** | 67.793 |
| **Glysine** | **10.981** | ***** | 8.609 | 2.749 |  | 1.928 | 0.144 |  | 0.103 | **13.586** | ***** | 6.629 |
| Serine | 0.243 |  | 0.158 | 0.184 |  | 0.028 | **0.312** | ***** | 0.117 | 0.115 |  | 0.039 |
| **Threonine** | **1.394** | ***** | 0.586 | 0.885 |  | 0.391 | 0.538 |  | 0.146 | **1.741** | ***** | 0.344 |
| beta_Alanine | 1.348 |  | 0.283 | **11.801** | ***** | 2.787 | 6.71 |  | 4.768 | 6.439 |  | 4.226 |
| Aspartic acid | 0.726 |  | 0.616 | **1.949** | ***** | 0.556 | 0.94 |  | 0.884 | 1.735 |  | 0.408 |
| **GABA** | 0.051 |  | 0.046 | **0.845** | ***** | 0.646 | 0.038 |  | 0.0283 | **0.857** | ***** | 0.637 |
| Oxoproline | 14.177 |  | 11.227 | 10.701 |  | 8.196 | 0.092 |  | 0.079 | **24.786** | ***** | 3.727 |
| Cysteine | 3.286 |  | 5.512 | 28.033 |  | 33.113 | 0.552 |  | 0.347 | 30.768 |  | 32.195 |
| **Arginine** | **78.883** | ***** | 66.536 | 3.69 |  | 3.368 | 0.536 |  | 0.359 | **82.037** | ***** | 64.356 |
| **Glutamine** | 11.695 |  | 9.389 | **40.584** | ***** | 32.509 | 0.016 |  | 0.001 | **52.263** | ***** | 24.01 |
| Glutamic acid | 0.542 |  | 0.372 | 0.681 |  | 0.258 | 0.226 |  | 0.131 | **0.998** | ***** | 0.124 |
| **Phenylalanine** | 28.002 |  | 21.976 | **60.019** | ***** | 47.709 | 0.646 |  | 0.484 | **87.375** | ***** | 28.457 |
| **Asparagine** | 5.697 |  | 3.266 | **36.347** | ***** | 29.267 | 1.612 |  | 0.134 | **40.432** | ***** | 26.399 |
| **Lysine** | 23.286 |  | 18.491 | **262.656** | ***** | 214.96 | 0.52 |  | 0.372 | **285.42** | ***** | 198.46 |
| **Tyrosine** | **604.147** | ***** | 484.79 | 191.657 |  | 165.04 | 1 |  | 0 | **794.8** | ***** | 352.51 |
| **Tryptophan** | 1 |  | 0 | **47.883** | ***** | 38.565 | 1 |  | 0 | **47.883** | ***** | 38.565 |
| **Total** | 3.483 |  | 2.456 | 3.062 |  | 2.118 | 0.353 |  | 0.061 | **6.193** | ***** | 0.582 |
| **Other compounds** | | | | | | | | | | | | |
| **Ethanolamine** | 2.01 |  | 0.869 | **3.625** | ***** | 2.435 | 0.738 |  | 0.17 | **4.897** | ***** | 1.5 |
| Phosphoric acid | 3.479 |  | 2.754 | 2.831 |  | 1.895 | 0.226 |  | 0.201 | **6.085** | ***** | 0.982 |
| **Nicotine** | 1.546 |  | 0.317 | **10.559** | ***** | 3.08 | **7.656** | ***** | 4.802 | 4.449 |  | 2.796 |
| Putrescine | 0.226 |  | 0.04 | 0.178 |  | 0.065 | 0.166 |  | 0.041 | 0.238 |  | 0.058 |
| **Pantothenate** | **81.942** | ***** | 65.554 | 20.294 |  | 15.938 | 1 |  | 0 | **101.24** | ***** | 51.742 |
| Purine | 66.974 |  | 61.402 | 44.285 |  | 34.804 | 1 |  | 0 | **110.26** | ***** | 39.43 |
| **Phenylethanolamine** | **57.943** | ***** | 45.42 | 20.726 |  | 15.489 | 1 |  | 0 | **77.669** | ***** | 30.9 |
| **Total** | 1.245 |  | 0.636 | **2.158** | ***** | 0.703 | 0.856 |  | 0.355 | **2.548** | ***** | 0.406 |

**Table S6**: Raw data of the primary metabolites of *Pvgstu*3-3.4 and WT plants under combined D_T stress (9 days) compared to control conditions at 0 days. Mean values of three independent determinations for each treatment were expressed as relative abundance compared to internal standard adonitol. Significant differences between genotypes for each treatment are indicated with different letters at p<0.05.

|  | **0d** | | | ***Pvgstu3-3.4* 9d** | | | | | | | **0d** | | | | **WT 9d** | | | | | | | |  | | |
| --- | --- | --- | --- | --- | --- | --- | --- | --- | --- | --- | --- | --- | --- | --- | --- | --- | --- | --- | --- | --- | --- | --- | --- | --- | --- |
| **Metabolites** | ***Pvgstu3-3.4*** | | | **Control** | | | **DT** | | | | **WT** | | | | **Control** | | | | **DT** | | | |  |  |  |
|  | **Mean** |  | **±SE** | **Mean** |  | **±SE** | **Mean** |  | **±SE** | **Mean** | |  | **±SE e** | **Mean** | |  | **±SE** | **Mean** | |  | **±SE** | **p-value** | |  |  |
| **Soluble sugars** | | | | | | | | | | | | | | | | | | | | | | | | | |
| Xylose | 0.075 | c | 0.014 | 0.065 | c | 0.013 | 0.615 | a | 0.079 | 0.079 | | c | 0.020 | 0.059 | | c | 0.011 | 0.268 | | b | 0.068 | 0.000006 | | |  |
| Arabinose | 0.016 | d | 0.001 | 0 | e | 0 | 0.059 | b | 0 | 0.018 | | d | 0.002 | 0.03 | | c | 0.005 | 0.099 | | a | 0.007 | <0.05 | | |  |
| Threose | 0 | c | 0 | 0 | c | 0 | 0.023 | bc | 0.005 | 0 | | c | 0 | 0.046 | | b | 0.004 | 0.09 | | a | 0.024 | 0.00017 | | |  |
| Fructose | 1.971 | c | 0.31 | 0.561 | c | 0.050 | 112.9 | a | 5.353 | 1.872 | | c | 0.561 | 1.416 | | c | 0.189 | 16.923 | | b | 1.392 | <0.05 | | |  |
| Glucose | 1.403 | c | 0.071 | 0.484 | c | 0.082 | 114.06 | a | 1.687 | 1.039 | | c | 0.275 | 1.561 | | c | 0.194 | 16.758 | | b | 1.967 | <0.05 | | |  |
| Galactose | 0.047 | c | 0.023 | 0 | c | 0 | 1.378 | a | 0.097 | 0 | | c | 0 | 0 | | c | 0 | 0.705 | | b | 0.097 | <0.05 | | |  |
| Erythrose | 0 | c | 0 | 0 | c | 0 | 0.031 | a | 0.007 | 0 | | c | 0 | 0 | | c | 0 | 0.014 | | b | 0.004 | 0.000226 | | |  |
| Mannose-6-deoxy | 0 | c | 0 | 0 | c | 0 | 3.684 | a | 0.274 | 0 | | c | 0 | 0 | | c | 0 | 1.209 | | b | 0.336 | <0.05 | | |  |
| Sucrose | 16.207 | cd | 2.991 | 21.382 | bc | 2.634 | 44.02 | a | 2.59 | 13.045 | | d | 1.233 | 27.71 | | b | 2.532 | 13.594 | | cd | 2.113 | 0.000007 | | |  |
| Total | 19.718 | cd | 2.962 | 22.493 | cd | 2.678 | 276.77 | a | 7.863 | 16.053 | | d | 1.27 | 30.822 | | c | 2.538 | 49.66 | | b | 5.567 | <0.05 | | |  |
| **Soluble alcohols** | | | | | | | | | | | | | | | | | | | | | | | | | |
| Glycerol | 0.334 | c | 0.071 | 0.316 | c | 0.057 | 0.222 | c | 0.011 | 0.672 | | b | 0.030 | 0.196 | | c | 0.044 | 0.833 | | a | 0.046 | 0.000002 | | |  |
| Meso_eryrthitol | 0.027 | c | 0.007 | 0 | c | 0 | 0.099 | b | 0.013 | 0.03 | | c | 0.003 | 0.127 | | ab | 0.046 | 0.171 | | a | 0.009 | 0.000352 | | |  |
| Mannitol | 0.044 | d | 0.007 | 0 | d | 0 | 1.362 | a | 0.061 | 0.021 | | d | 0.005 | 0.244 | | c | 0.031 | 0.4 | | b | 0.103 | <0.05 | | |  |
| Sorbitol | 0 | c | 0 | 0 | c | 0 | 0.293 | a | 0.052 | 0 | | c | 0 | 0.032 | | bc | 0.004 | 0.094 | | b | 0.022 | 0.000006 | | |  |
| Myoinisitol | 3.976 | b | 0.403 | 3.892 | b | 0.274 | 23.04 | a | 2.958 | 4.28 | | b | 0.797 | 23.411 | | a | 3.359 | 6.414 | | b | 0.683 | 0.000005 | | |  |
| Total | 4.38 | b | 0.348 | 4.207 | b | 0.296 | 25.017 | a | 2.965 | 5.002 | | b | 0.772 | 24.011 | | a | 3.368 | 7.913 | | b | 0.815 | 0.000003 | | |  |
| **Organic acids** | | | | | | | | | | | | | | | | | | | | | | | | | |
| Glyceric acid | 0.016 | c | 0.003 | 0 | c | 0 | 0.234 | a | 0.064 | 0.071 | | bc | 0.013 | 0.153 | | ab | 0.0306 | 0.256 | | a | 0.066 | 0.00209 | | |  |
| Malic acid | 0.026 | b | 0.006 | 0.084 | b | 0.021 | 0.465 | b | 0.076 | 0.035 | | b | 0.002 | 1.162 | | a | 0.205 | 1.209 | | a | 0.364 | 0.000508 | | |  |
| Threonic acid | 0.036 | c | 0.011 | 0.096 | c | 0.004 | 0.703 | a | 0.106 | 0.077 | | c | 0.036 | 0.283 | | b | 0.047 | 0.45 | | b | 0.058 | 0.000011 | | |  |
| 2-Oxoglutaric acid | 0 | b | 0 | 0 | b | 0 | 0.182 | a | 0.063 | 0.004 | | b | 0.005 | 0 | | b | 0 | 0.162 | | a | 0.026 | 0.000678 | | |  |
| Citric acid | 0 | b | 0 | 0 | b | 0 | 0.054 | a | 0.032 | 0 | | b | 0 | 0 | | b | 0 | 0.047 | | a | 0.004 | 0.027926 | | |  |
| Quinic acid | 0.069 | a | 0.009 | 0.142 | a | 0.005 | 0.116 | a | 0.048 | 0.107 | | a | 0.031 | 0.103 | | a | 0.021 | 0.102 | | a | 0.015 | 0.563954 | | |  |
| Gluconic acid | 0.018 | bc | 0.002 | 0 | c | 0 | 0.085 | a | 0.021 | 0.026 | | bc | 0.005 | 0 | | c | 0 | 0.036 | | b | 0.009 | 0.000467 | | |  |
| 2-Aminoadipic acid | 0 | b | 0 | 0 | b | 0 | 0.042 | a | 0.012 | 0 | | b | 0 | 0 | | b | 0 | 0.049 | | a | 0.013 | 0.000422 | | |  |
| Total | 0.165 | b | 0.013 | 0.322 | b | 0.019 | 1.839 | a | 0.245 | 0.321 | | b | 0.084 | 1.701 | | a | 0.244 | 2.261 | | a | 0.519 | 0.000163 | | |  |
| **Amino acids** | | | | | | | | | | | | | | | | | | | | | | | | | |
| Alanine | 0.102 | b | 0.025 | 0.068 | b | 0.007 | 0.048 | b | 0.006 | 0.099 | | b | 0.019 | 0.092 | | b | 0.033 | 0.317 | | a | 0.071 | 0.001614 | | |  |
| Valine | 0.069 | c | 0.010 | 0.001 | c | 0 | 0.621 | b | 0.033 | 0.089 | | c | 0.014 | 0.049 | | c | 0.006 | 1.123 | | a | 0.154 | <0.05 | | |  |
| Isoleucine | 0.038 | b | 0.01 | 0 | b | 0 | 0.458 | a | 0.043 | 0.057 | | b | 0.011 | 0.025 | | b | 0.006 | 0.394 | | a | 0.099 | 0.000014 | | |  |
| Proline | 0.056 | c | 0.006 | 0 | c | 0 | 16.62 | a | 1.195 | 0.096 | | c | 0.006 | 0.049 | | c | 0.006 | 12.204 | | b | 1.373 | <0.05 | | |  |
| Glysine | 0.091 | c | 0.003 | 0 | c | 0 | 2.001 | a | 0.163 | 0.157 | | c | 0.005 | 0.044 | | c | 0.0006 | 0.822 | | b | 0.046 | <0.05 | | |  |
| Serine | 3.123 | a | 0.161 | 1.313 | b | 0.318 | 0.205 | c | 0.022 | 3.48 | | a | 0.234 | 0.711 | | c | 0.103 | 0.573 | | c | 0.03 | <0.05 | | |  |
| Threonine | 0.463 | bc | 0.026 | 0.307 | cd | 0.028 | 0.983 | a | 0.087 | 0.431 | | bc | 0.008 | 0.178 | | d | 0.054 | 0.585 | | b | 0.053 | 0.000002 | | |  |
| beta_Alanine | 0.078 | b | 0.017 | 0.084 | b | 0.005 | 0.125 | b | 0.019 | 0.046 | | b | 0.005 | 0.566 | | a | 0.115 | 0.517 | | a | 0.116 | 0.000208 | | |  |
| Aspartic acid | 0.041 | a | 0.008 | 0 | b | 0 | 0.059 | a | 0.015 | 0.034 | | ab | 0.005 | 0.062 | | a | 0.023 | 0.069 | | a | 0.007 | 0.020356 | | |  |
| GABA | 0.295 | b | 0.087 | 0 | c | 0 | 0.029 | c | 0.011 | 0.362 | | ab | 0.140 | 0.026 | | c | 0.004 | 0.585 | | a | 0.114 | 0.001332 | | |  |
| Oxoproline | 0.311 | c | 0.065 | 0 | c | 0 | 8.807 | a | 0.942 | 0.304 | | c | 0.025 | 0.055 | | c | 0.015 | 6.456 | | b | 0.343 | <0.05 | | |  |
| Cysteine | 0.009 | a | 0.01 | 0 | a | 0 | 0.061 | a | 0.061 | 0 | | a | 0 | 0 | | a | 0 | 0.055 | | a | 0.03 | 0.446639 | | |  |
| Arginine | 0 | b | 0 | 0 | b | 0 | 0.157 | a | 0.036 | 0.013 | | b | 0.013 | 0 | | b | 0 | 0.101 | | a | 0.033 | 0.00049 | | |  |
| Glutamine | 0.072 | c | 0.020 | 0 | c | 0 | 1.692 | b | 0.234 | 0.055 | | c | 0.014 | 0 | | c | 0 | 4.436 | | a | 0.588 | <0.05 | | |  |
| Glutamic acid | 0.736 | a | 0.111 | 0.047 | c | 0.005 | 0.751 | a | 0.033 | 0.843 | | a | 0.121 | 0.327 | | b | 0.043 | 0.822 | | a | 0.127 | 0.000133 | | |  |
| Phenylalanine | 0.03 | c | 0.004 | 0 | c | 0 | 1.666 | b | 0.142 | 0.029 | | c | 0.002 | 0.037 | | c | 0.003 | 3.462 | | a | 0.538 | <0.05 | | |  |
| Asparagine | 0.029 | b | 0.006 | 0.045 | b | 0.002 | 0.288 | b | 0.021 | 0.025 | | b | 0.005 | 0.042 | | b | 0.003 | 1.752 | | a | 0.371 | 0.000017 | | |  |
| Lysine | 0.025 | c | 0.002 | 0 | c | 0 | 1.149 | a | 0.134 | 0 | | c | 0 | 0 | | c | 0 | 0.524 | | b | 0.092 | <0.05 | | |  |
| Tyrosine | 0 | c | 0 | 0 | c | 0 | 1.207 | a | 0.167 | 0 | | c | 0 | 0 | | c | 0 | 0.382 | | b | 0.095 | 0.000001 | | |  |
| Tryptophan | 0 | b | 0 | 0 | b | 0 | 0 | b | 0 | 0 | | b | 0 | 0 | | b | 0 | 0.072 | | a | 0.027 | 0.000002 | | |  |
| Total | 5.56 | b | 0.334 | 1.866 | b | 0.346 | 36.86 | a | 2.127 | 6.12 | | b | 0.355 | 2.262 | | b | 0.272 | 35.217 | | a | 2.915 | <0.05 | | |  |
| **Other compounds** | | | | | | | | | | | | | | | | | | | | | | | | | |
| Ethanolamine | 0.118 | c | 0.008 | 0.11 | c | 0.007 | 0.365 | b | 0.036 | 0.13 | | c | 0.009 | 0.071 | | c | 0.011 | 0.874 | | a | 0.081 | <0.05 | | |  |
| Phosphoric acid | 0.147 | cd | 0.013 | 0 | d | 0 | 1.019 | b | 0.112 | 0.254 | | c | 0.008 | 0.113 | | cd | 0.036 | 1.327 | | a | 0.132 | <0.05 | | |  |
| Nicotine | 0.113 | c | 0.026 | 0.213 | c | 0.019 | 0.138 | c | 0.018 | 0.079 | | c | 0.0075 | 1.063 | | a | 0.177 | 0.608 | | b | 0.126 | 0.000026 | | |  |
| Putrescine | 0.665 | a | 0.169 | 0.121 | b | 0.017 | 0.18 | b | 0.005 | 0.573 | | a | 0.027 | 0.087 | | b | 0.025 | 0.117 | | b | 0.038 | 0.000205 | | |  |
| Pantothenate | 0 | c | 0 | 0 | c | 0 | 0.163 | a | 0.025 | 0 | | c | 0 | 0 | | c | 0 | 0.04 | | b | 0.007 | 0.000001 | | |  |
| Purine | 0 | b | 0 | 0 | b | 0 | 0.133 | a | 0.044 | 0 | | b | 0 | 0 | | b | 0 | 0.088 | | a | 0.012 | 0.000646 | | |  |
| Phenylethanolamine | 0 | c | 0 | 0 | c | 0 | 0.115 | a | 0.014 | 0 | | c | 0 | 0 | | c | 0 | 0.04 | | b | 0.003 | <0.05 | | |  |
| Total | 1.044 | c | 0.188 | 0.444 | d | 0.035 | 2.154 | b | 0.055 | 1.037 | | c | 0.038 | 1.334 | | c | 0.159 | 3.143 | | a | 0.203 | <0.05 | | |  |
